# In silico identification of multiple conserved motifs within the control region of Culicidae mitogenomes

**DOI:** 10.1038/s41598-022-26236-5

**Published:** 2022-12-19

**Authors:** Thomas M. R. Harrison, Josip Rudar, Nicholas Ogden, Royce Steeves, David R. Lapen, Donald Baird, Nellie Gagné, Oliver Lung

**Affiliations:** 1 Canadian Food Inspection Agency, National Centre for Foreign Animal Disease, 1015 Arlington St. Winnipeg, Manitoba, R3M 3E4 Canada; 2 Public Health Risk Sciences Division, National Microbiology Laboratory, Public Health Agency of Canada, Saint-Hyacinthe, QC Canada; 3 Gulf Fisheries Centre, Fisheries & Oceans Canada, Moncton, New Brunswick, Canada; 4 Ottawa Research Development Centre, Agriculture & Agri-Food Canada, Ottawa, ON K1A 0C6 Canada; 5 Environment and Climate Change Canada, Canadian Rivers Institute, Department of Biology, University of New Brunswick, Fredericton, NB Canada; 6 Department of Biological Sciences, University of Manitoba, Winnipeg, MB Canada

**Keywords:** Classification and taxonomy, Machine learning, Comparative genomics

## Abstract

Mosquitoes are important vectors for human and animal diseases. Genetic markers, like the mitochondrial COI gene, can facilitate the taxonomic classification of disease vectors, vector-borne disease surveillance, and prevention. Within the control region (CR) of the mitochondrial genome, there exists a highly variable and poorly studied non-coding AT-rich area that contains the origin of replication. Although the CR hypervariable region has been used for species differentiation of some animals, few studies have investigated the mosquito CR. In this study, we analyze the mosquito mitogenome CR sequences from 125 species and 17 genera. We discovered four conserved motifs located 80 to 230 bp upstream of the 12S rRNA gene. Two of these motifs were found within all 392 *Anopheles* (*An*.) CR sequences while the other two motifs were identified in all 37 *Culex* (*Cx*.) CR sequences. However, only 3 of the 304 non-Culicidae Dipteran mitogenome CR sequences contained these motifs. Interestingly, the short motif found in all 37 *Culex* sequences had poly-A and poly-T stretch of similar length that is predicted to form a stable hairpin. We show that supervised learning using the frequency chaos game representation of the CR can be used to differentiate mosquito genera from their dipteran relatives.

## Introduction

Mosquitos are a diverse group of over 3500 species belonging to the family Culicidae and roughly 100 species are known to be important vectors of human and animal disease^1^. The spread of mosquito-borne diseases (MBDs) exerts an enormous human and economic cost each year. In 2019 alone, the spread of malaria by the *Anopheles (An.) gambiae* species complex resulted in an estimated 228 million cases and 405 thousand deaths while Dengue fever virus, which is spread by *Aedes* (*Ae*.) (*Stegomyia) aegypti*, may have caused 390 million infections, with 96 million manifesting either at clinical or sub-clinical levels^1–3^. Other MBDs, such as Lymphatic filariasis, spread when members of *Culex* spp. carrying larval filarial worms bite a suitable host. This disease affects an estimated 120 million people worldwide and puts a further 1.1 billion at risk^4^. In addition, other less common but important pathogens such as yellow fever virus, Zika virus, West Nile virus, Rift Valley fever virus (RVFV), Western equine encephalitis virus, and Eastern equine encephalitis virus are spread by mosquitoes^3, 5–8^.

Due to their important role as human and animal disease vectors, clear identification of different species within the Culicidae could have broad implications in public health and our understanding of the spread of mosquito-borne diseases. This line of work is particularly important since increased globalization, urbanization, and climate change are predicted to boost the incidence of mosquito-borne diseases by influencing how pathogens are transmitted or changing the distribution and abundance of mosquito species and potential pathogen reservoirs^9^. Mitochondrial genomes (mitogenomes) are often used for phylogenetics, forensics, and species identification^10, 11^. The mitogenome is useful for these purposes since it is small, abundant, passed down maternally, and rarely recombines^12, 13^. The application of molecular and computational techniques to the analysis of this data can be particularly advantageous since these methods can be used to quickly, objectively, and efficiently analyze mitogenomes. Furthermore, bulk samples can contain information about a wider variety of species, and the analysis of this data does not require the services of an entomologist. If the costs are amortized over a large number of samples, molecular approaches can also be quicker, cheaper, and more consistent than morphological identification^14^.

The mosquito mitogenome is usually 14–20 kbp in length and contains 22 tRNA genes, 2 rRNA genes, and 13 protein-coding genes involved in the electron transport chain of oxidative phosphorylation^12, 13^. The control region (CR) of the mitogenome, which is investigated in this work, is an A + T-rich region which contains the hypervariable region and the origin of replication^15^. Depending on the taxonomic group, the boundary of the CR can differ. For example, in Hymenoptera, the CR is bounded by the tRNA^Glu^ and 12S rRNA genes while in Lepidoptera it is bounded by the tRNA^Met^ and 12S rRNA genes. In Dipterans, such as mosquitos, it is bounded by tRNA^Ile^ and 12S rRNA genes^16^. In insects, the major strand’s origin of replication is associated with a poly-T stretch^17^. In contrast, replication of the minor strand is thought to begin in or near a hypothesized hairpin stem-loop structure^18^. Investigations of the CR in mitogenomes from *An. gambiae* and *An. albitarsis* identified three conserved regions containing poly-T and poly-A stretches of 5–20 bp^16, 19–21^. However, beyond these investigations there has been no detailed comparative analysis of Culicidae CR sequences.

Of the 13 protein-coding genes, cytochrome oxidase I (COI) is particularly useful for taxonomic identification since it has been developed as a universal DNA barcode^22, 23^. Typically, a classifier (such as the RDP Classifier) is trained on the k-mer frequencies of the barcode region. When a new sample is presented to the classifier a taxonomic label is created by comparing the frequency of k-mers in the sample to that of the training dataset^24^. Recent work has demonstrated that transforming nucleic acid sequences into signatures can result in the development of successful machine learning classifiers^25, 26^. In this work, we continue to build upon this knowledge by using a type of genomic signature known as a frequency chaos game representation (FCGR) to train a semi-supervised deep learning model^27, 28^. An FCGR maps each nucleic acid sequences onto a unit square with the intensity of specific regions in the square corresponding to the frequency of *k* sized k-mers. This transformation preserves the information within each sequence while allowing for more interesting patterns in the distribution of frequencies to be analyzed by more complex machine learning models, such as neural networks^25, 26, 29^. While not as thoroughly investigated as COI, the CR has also shown potential as a marker for species identification^13, 30, 31^. In this work, we conduct an in silico investigation into 472 publicly available mosquito mitogenome sequences. Our goals are to identify conserved features within Culicidae CRs and investigate, using a deep-learning model, if this region can be used to accurately classify different mosquito genera.

## Methods

### Sequence data collection

A total of 813 sequences were retrieved from NCBI GenBank after searching for Culicidae with a mitochondrial source and a length of 12 to 21 kb, on May 15th, 2022. Sequences were removed from downstream analysis using the following quality control criteria: lack of a CR, or CR under 200 bp (n = 202); ambiguous nucleotides within the CR (n = 82); unverified origin species (including nr. and aff. designations) (n = 46). Three unannotated genomes (OU632726, MH316118, MH316119) were annotated using MITOS2 (Revision 999) (http://mitos2.bioinf.uni-leipzig.de/index.py). Geneious (version R11.1) (https://www.geneious.com/) was used to extract the CRs from each sequence using tRNA^Ile^ and 12S rRNA annotations as the boundaries. A total of 472 sequences representing 125 species from 17 genera were included in the final analysis (Table S1).

### Motif discovery and annotation

The MEME Suite (version 5.0.5) (http://meme-suite.org/)^32^ was used for motif discovery and search on the 472 sequences retrieved, and it assigns each discovered motif an E-value. MEME was used on the 472 sequences with the following flags and options. With *Aedes* CR sequences MEME was run with the ‘-dna’ and ‘-mod anr’ options. These options allow the software to search DNA sequences for any number of repeats. To prevent unnecessary use of computational resources, searches were stopped when the E-value of a motif was greater than 1000 (the ‘-evt’ parameter) or when MEME identified at least 100 motifs. The maximum width of each motif was set to be 20 base pairs. The p-value of each motif was estimated using the Hertz and Stormo’s Numerically Correct algorithm. Two Anopheles CR sequences were not used, as they were missing a region beginning with at least 3 cytosines and at least 49 bp long: MK575478 (*An. darlingi*), and MK575477 (*An. cruzii*). Additional CR sequences of these species are represented elsewhere in the dataset. One *Culex* sequence (MK575480 (*Cx. quinquefasciatus*)) was not used since the CR for this sequence did not contain a run of 4–6 cytosines. This species is represented elsewhere in the dataset.

The maximum width of motifs in *Anopheles* and *Culex* sequences was set to 25 and the ‘-**mod** oops’ option, which forces MEME to find one occurrence per sequence for each motif, was used. Use of ‘-mod oops’ with *Aedes* sequences did not give results with good sequence conservation due to the low number and high diversity of *Aedes* sequences. To confirm the presence of the motifs, a MAST analysis was performed using all the short motifs discovered by MEME (32). All 473 mosquito mitogenomes were used in this analysis using the ‘**-bfile** –motif–' option to correct for the nucleotide frequency of the CR. This is necessary because MAST uses nucleotide frequencies that are derived from a non-redundant DNA database that does not have a similar profile of nucleotide frequencies as the CR. If left uncorrected, this causes MAST to assume that long stretches of A and T are more significant than they are in the CR, where the AT content is approximately 90%.

**Figure 1:**
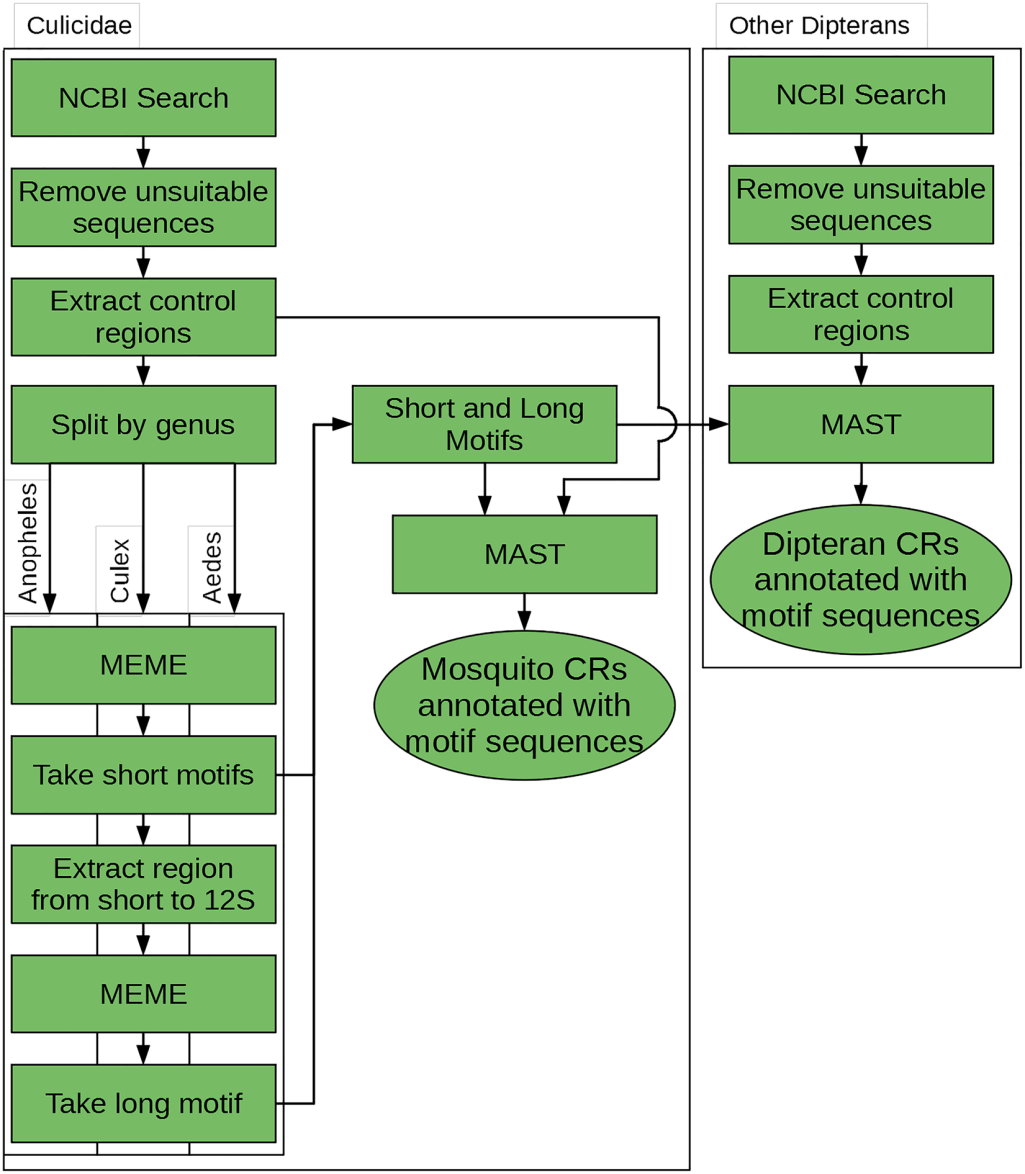
Overview flowchart showing the data processing. *Anopheles*, *Culex*, and *Aedes* sequences were treated separately, but used the same procedure.

To locate the long motifs, the region between the end of the 12S rRNA gene and the short motif was extracted and split into groups by genus. MEME was run using the following options: ‘-**dna** -**evt** 1000 -**mod** anr -**nmotifs** 100 -**maxw** 50’. This produced a set of longer motifs: *Anopheles* Long Motif (AnLM), and *Culex* Long Motif (CLM). The Aedes sequences did not produce any motifs that were present on >  = 90% of sequences tested, so there was no “long” motif for *Aedes*. These long motifs and the short motifs were searched for in the original 472 sequences with MAST, using the “-**bfile** –motif–” option. An overview of the CR motif discovery and annotation workflow is presented in Fig. 1.

### Culex DNA hairpin simulation

DNA folding simulation and visualization were performed with 27 sequences from the *Culex* mitogenomes including and flanking the CSM identified by MEME (15 bp of the upstream poly-A region and 15 bp downstream of the guanine after the poly-T region) using the DNA profile of RNAfold (version 2.4.12) from the ViennaRNA software package (https://almob.biomedcentral.com/articles/10.1186/1748-7188-6-26). The DNA profile disabled conversion of T to U (“-P "DNA" –noconv”). Each of the 27 sequences was analyzed at increments of 5 °C from 10 to 50 °C.

### Validation against non-Culicidae dipteran mitogenomes

A total of 824 non-Culicidae Dipteran mitogenomes were retrieved from NCBI for comparison with CR sequences from Culicidae. Sequences were excluded from the downstream analysis based on the following criteria: incomplete mitogenome annotation or missing CR boundaries (n = 109); unverified species (n = 28); CR less than 150 bp or missing (n = 352); contain ambiguous nucleotides in CR sequence (n = 30). Of the 824 mitogenome sequences, 304 passed the selection criteria (Table S2). Short and Long motifs in these 304 sequences were located with MAST with the “**-bfile** –motif–” option.

In addition to the above analysis, we investigated if semi-supervised machine learning could learn to differentiate between the CRs of Culicidae and non-Culicidae mitogenomes. CRs from the dataset used for the motif investigation were first filtered to remove sequences containing ambiguous nucleotides. This was done since FCGRs are usually unable to handle ambiguous nucleotides. To train a more stable model, species which occur 5 or fewer times in the dataset were also removed and the resulting set of sequences was de-duplicated to ensure that it only contained unique CRs. Sequences were then reverse complemented. The original sequence and its reverse complement were then concatenated^33^. This resulted in a final set of 673 Culicidae and non-Culicidae sequences. Following this, each CR sequence was transformed into a FCGR with a depth of 6^29^. Previous work has shown that this representation has the ability to efficiently summarize the unique characteristics of each genome and can be used for clustering and supervised learning^26, 34–36^. The FCGRs for each CR, along with the class labels, were then used as inputs into a semi-supervised deep learning model^27^. A brief overview of this model is presented in Fig. 4.

This model is a self-supervised generative adversarial network (SGAN) in which two networks, the discriminator and generator, compete against each other so that a good representation of real data (in this case, FCGR representations of CRs) can be learned^27^. The discriminator network is itself an ensemble of two branches, each of which attempts to learn a different set of reduced features that can be used to classify FCGRs from each class (*Aedes*, *Anopheles*, *Culex*, and Non-Culicidae Dipterans) and (during training) if a sample is real or synthetic. The first and second branch of the discriminator network is based on vision transformer and FNet encoder, respectively^37, 38^. The generator network learns to create FCGRs that are become increasingly difficult to distinguish from real FCGRs. Before training the model, random oversampling of each minority class was performed on the input data to ensure that the remaining under-represented classes contained at least 50 samples. This was done using the ‘imbalanced-learn’ (version 0.8.1) Python package^39^.

For each path in the discriminator, a separate set of non-overlapping 4 × 4 patches of each FCGR were created^37, 40, 41^. Learned positional embeddings are not added to the patches. This results in 256 16-dimensional patches (the patches are flattened). A non-linear projection of these patches is then created using the embedding block (Suppl Fig. 1)^37, 40^. Depending on the path, each embedding is passed to an attention block or a FNet block (Suppl Fig. 2)^37, 38^. Finally, a “summary” (a tensor of size batch size × 256) is created from the attention or FNet blocks by passing their output through a mixing block which make use of global average pooling layers (Suppl Fig. 3). The output of each branch’s mixing block is concatenated before being passed to a small feed forward network (FFN) with two outputs. One output classifies samples according to taxon and the second determines if the sample is real or synthetic (Suppl Fig. 4). All dense layers, except for the final layer, in the network use the ‘mish’ activation function^42^. The final layer uses a linear activation, and the output of this layer is used in conjunction with a SoftMax and custom activation (described in Salimans et al.) to classify samples and determine if the sample is real or synthetic, respectively^27, 43^. Dropout layers and Gaussian Noise are used in key areas of the discriminator network to prevent overfitting (Suppl Figs. 1–4)^44^.

The generator network begins by sampling from a random normal distribution to create a set of 160-dimensional vectors. These vectors are then projected into a higher dimensional space and reshaped into a tensor of size 8 × 8 × 256 (Suppl Fig. 5). Three sets of convolutions, up-sample each 8 × 8 × 256 tensor into a 64 × 64 × 32 tensor. A final one-dimensional convolution across the channel dimension followed by a ReLU activation layer (since FCGRs have no negative components) creates the final set of synthetic FCGRs. Dropout layers are used in the key areas of the generator network to prevent overfitting^44^.

The final model is a meta-estimator created from ensemble of twenty different networks, each trained on different instances of the original training data and using different weight initializations^45^. The GAN itself uses the generator output (a synthetic FCGR) as its input and the unsupervised discriminator model as its output^43^. When the generator is being updated during training, the weights of the discriminator model are not trainable. Algorithm One outlines the basic steps for training the SGAN. Finally, after each model in the ensemble is trained only the classification portion of the discriminator is used for inference with the predictions from each classification model being averaged to determine the final prediction. Lookahead optimization using the AdamW optimizer and gradient centralization was used. The ‘weight_decay’ parameter was set to 1 × 10^–6^ while the ‘beta_1’ parameter was set to 0.5^46–48^. The discriminator model contains 879,172 parameters while the generator model contains 3,376,129 parameters. The full code of the model is available at https://github.com/jrudar/In-Silico-Identification-of-Multiple-Conserved-Motifs-Within-the-CR-of-Culicidae-Mitogenomes.
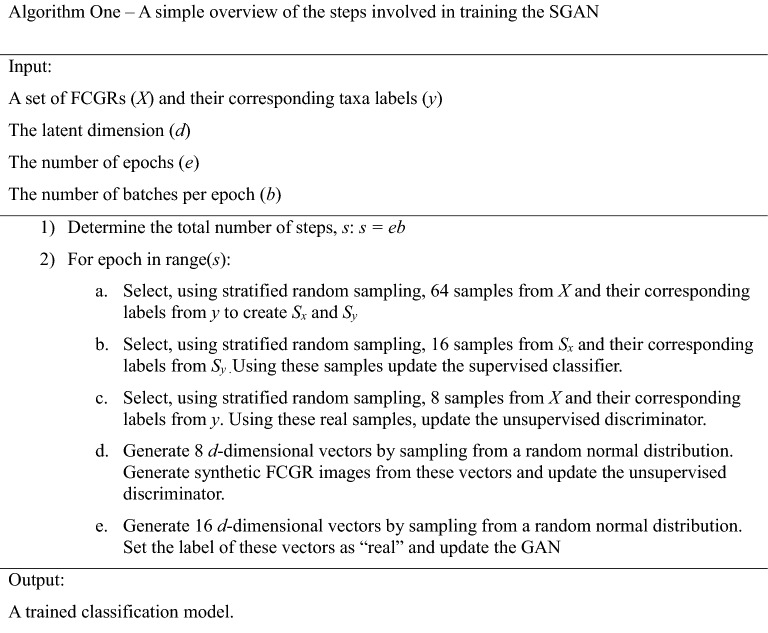


Next, we tested the generalization performance of our model. Data was divided into two groups, sequences sampled and assembled in 2019 and those assembled afterward, and these groups were used to assess how well the model performed. Five-fold cross-validation was used to test the first condition while Five-fold repeated cross-validation using 5 repeats was used to test the second^49^. The Smooth-Grad implementation found in the tf-keras-vis package (available at https://github.com/tf-keras-vis) was used to compute saliency maps for each sample in each class. To create the saliency maps, the noise parameter was set at 0.2 while the smoothing parameter was set at 30. The set of maps for each class were then averaged to create an average saliency map for each class. Finally, our model was compared to the Scikit-Learn implementation of the Extra Trees Classifier (using 512 trees), Logistic Regression (‘max_iter’ was set to 1000), and the Linear Support Vector Machine Classifier^49, 50^. Since our approach is semi-supervised, we also tested the performance of these models after wrapping them using Scikit-Learn’s implementation of the self-training classifier (the ‘k_best’ and ‘max_iter’ parameters were set to 5 and 100, respectively)^49, 51^. The labels for a random 40% of points in the training data were removed.

## Results

### Culicidae mitogenome attributes

**Figure 2:**
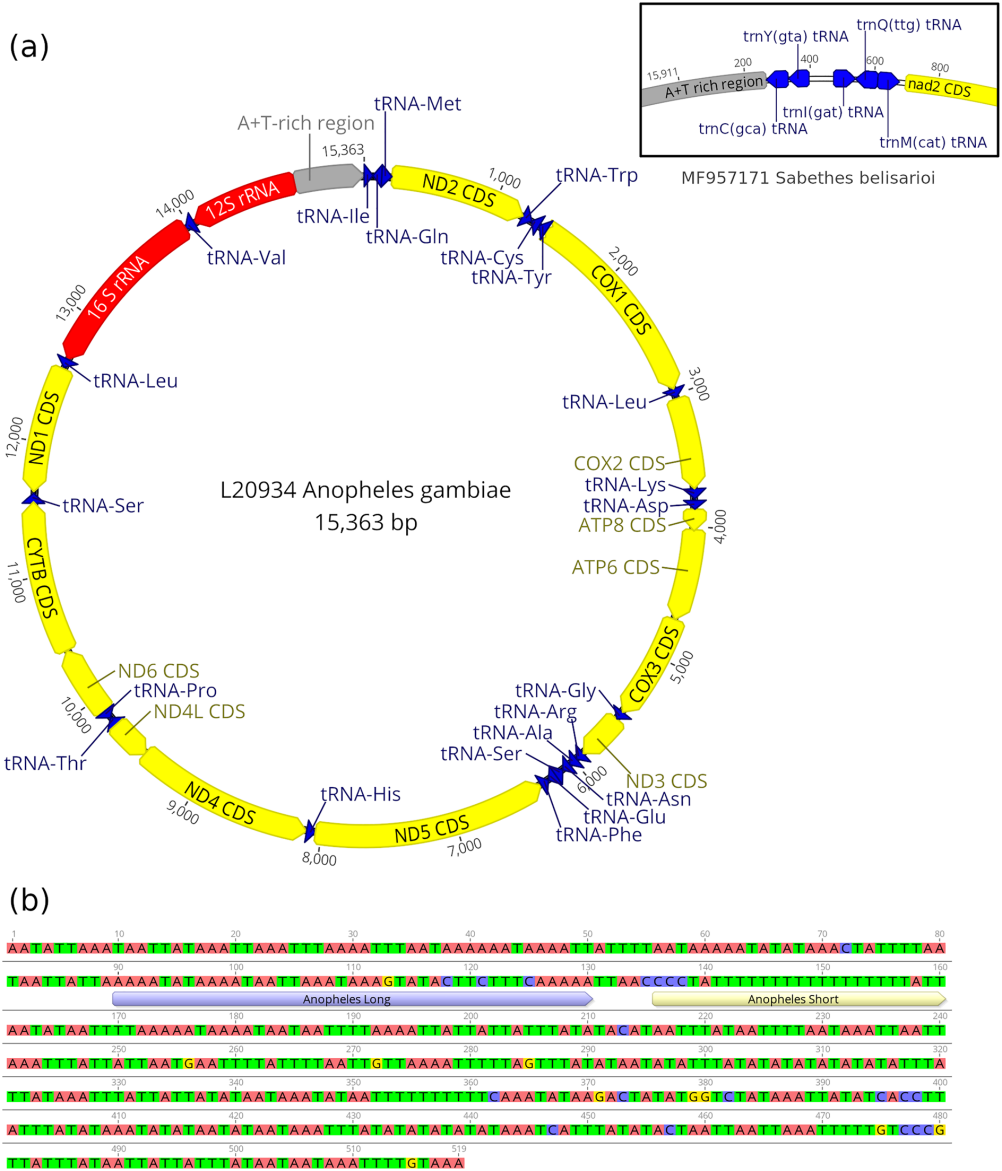
(**a**) Mitochondrial genome of *Anopheles gambiae* L20934. Yellow regions are the coding sequences of protein coding genes. Red regions are rRNA genes. Blue regions are tRNA genes. Grey region is the control region. Organization of genes is the same in all genera except *Sabethes*, where the tRNA^Cys^ and tRNA^Tyr^ genes are found in the control region, rather than between the ND2 and COX1 CDS. Gene organization for Sabethes belisarioi MF957171 tRNA bordering the control region is shown in an inset. (**b**) Close up of *Anopheles gambiae* L20934 control region with motif regions labelled. Generated with Geneious R11.1 (https://www.geneious.com).

A total of 472 mosquito mitogenomes from 125 species and 17 genera were analyzed in this study. The mean length of the mitogenomes for all sequences (n = 472), *Anopheles* (n = 392), *Culex* (n = 37), and *Aedes* (n = 16) were 15 461.5, 15 400.7, 15 574.0, and 16 378.0 bp, respectively. The mean %GC of the mitogenomes for all sequences, *Anopheles*, *Culex*, and *Aedes* was 22.0, 22.2, 21.4, and 20.8%, respectively (Table 1). All mitogenomes had the same gene order and orientation, except in *Sabethes*, *Runchomyia*, *Trichoprosopon*, *Tripteroides*, and *Wyeomyia* spp., where the tRNA^Cys^ and tRNA ^Tyr^ genes were located upstream of the tRNA^Ile^ gene on the majority strand (Fig. 2a). Both *Ae. alboannulatus* sequences in the dataset had the tRNA^Met^ and tRNA^Gln^ genes in reverse order. The mean length of the CR across all 472 mosquito mitogenome sequences was 611.1 bp. The mean CR length for *Anopheles*, *Culex*, and *Aedes* were 560.9, 711.7, and 1417.4 bp, respectively. The mean %GC of all CR was 7.4%. The mean %GC of *Anopheles*, *Culex*, and *Aedes* CR were 6.9, 10.5, and 7.5%, respectively (Table 2).

**Table 1:** Size and GC content of Culicidae mitogenomes by genus.

Genus	Mitogenome size (bp)			GC content		
	Mean	Minimum	Maximum	Mean (%)	Minimum	Maximum
All sequences (n = 472)	15,461.5	15,068 (MG816557 *Anopheles sinensis*)	17,150 (KX383916 Aedes *albopictus*)	22.0	19.3% (MF957171 *Sabethes belisarioi*)	24.6% (MF381635 *Anopheles parvus*)
*Anopheles (n* = *392)*	1,000.7	15,068 (MG816557 *Anopheles sinensis*)	15,734 (MF381706 *Anopheles eiseni*)	22.2	20.7% (MF381706 *Anopheles eiseni*)	24.6% (MF381635 *Anopheles parvus*)
*Culex (n* = *37)*	1,574.0	15,123 (KT852976 *Culex tritaeniorhynchus*)	16,052 (MF381720 *Culex chidesteri*)	21.4	20.4% (MF381720 *Culex chidesteri*)	22.3% (KT852976 *Culex tritaeniorhynchus*)
*Aedes (n* = *16)*	1,378.0	15,808 (MN389466 *Aedes rubrithorax*)	17,150 (KX383916 Aedes *albopictus*)	20.8	19.7% (KX383916 Aedes *albopictus*)	22.4% (MT093832 *Aedes koreicus*)

**Table 2:** Size and GC content of Culicidae mitogenome control region by genus.

Genus	Mitogenome size (bp)			GC Content		
	Mean	Minimum	Maximum	Mean (%)	Minimum	Maximum
All sequences	611.1	210 (MK575477 *Anopheles cruzii*)	2254 (KX383916 *Aedes albopictus*)	7.4	0.4% (MK575480 *Culex quinquefasciatus*)	12.3% (MF381722 *Culex bilineatus*)
*Anopheles*	560.9	210 (MK575477 *Anopheles cruzii*)	897 (MF381706 *Anopheles eiseni*)	6.9	2.1% (MK575478 *Anopheles darlingi*)	10.7% (MZ062478 *Anopheles janconnae*)
*Culex*	711.7	238 (MK575480 Culex quinquefasciatus)	1195 (MF381720 *Culex chidesteri*)	10.5	0.4% (MK575480 *Culex quinquefasciatus*)	12.3% (MF381722 *Culex bilineatus*)
*Aedes*	1417.4	748 (MN389466 Aedes rubrithorax)	2254 (KX383916 Aedes *albopictus*)	7.5	6.1% (KM676218 Aedes notoscriptus)	10.7% (MN389464 Aedes alboannulatus)

### Unique conserved motifs can be found in the Culicidae control region

A preliminary motif region, containing a poly-T stretch at the end of the motif, was found in the CR. Two conserved areas within this motif region were present, which we denote as “short” and “long” motifs. The short motifs were located near the poly-T stretch while the long motifs were found further upstream. Extraction and sorting of the preliminary motif region from each sequence by genus revealed that *Anopheles* motifs tended to begin with 4 cytosines which were then followed by 10–20 thymines. These motifs were located 130–160 bp from the end of the 12S rRNA. In *Culex*, the region started 12 bp upstream of the first cytosine in the run of 4–6 cytosines and was found 130–155 bp from the end of the 12S rRNA, after a stretch of 7–10 adenine residues. In *Aedes*, the region was started from the first occurrence of CCCTTAA from the end of the 12S rRNA. All extracted regions were 49 bp long. Each of these sets of regions were analyzed with MEME and are referred to as the *Anopheles* Short Motif (AnSM), *Culex* Short Motif (CSM), and *Aedes* Short Motif (AeSM).

**Figure 3:**
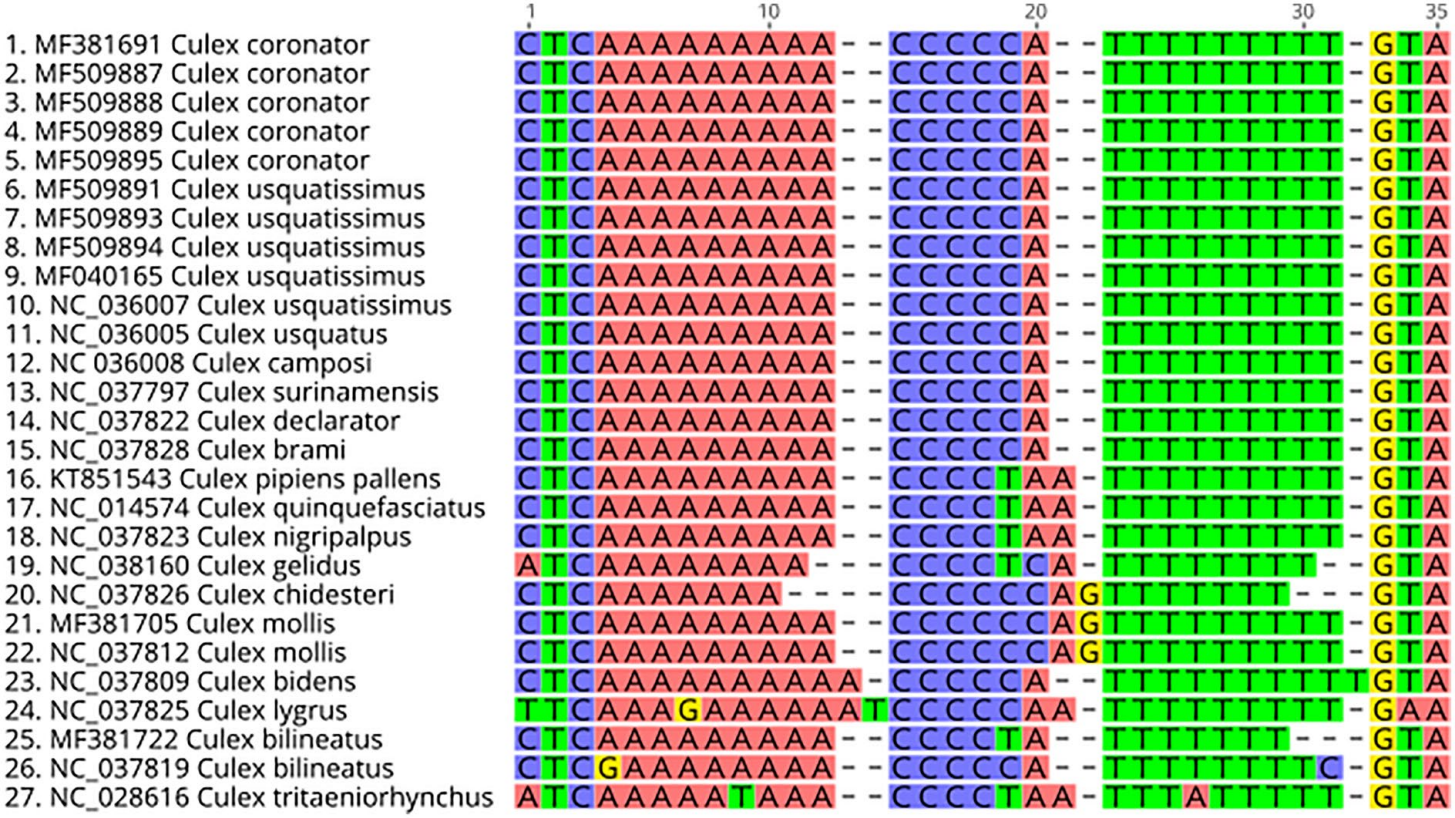
Comparison of 27 Culex Short motif and mirrored A and T stretches. Note mirrored mutations in 26. NC_037819 (G in position 4 and C in position 31) and 27. NC_028616 (T in position 9 and A in position 26). Generated with Geneious R11.1 (https://www.geneious.com).

Short motifs were found 129–172 bp upstream of the 12S rRNA gene in the MEME motif search (Table 3) while long motifs were detected roughly 76–105 bp upstream of the 12S rRNA gene (Table 3). Short motifs which appeared predominantly in *Anopheles* and *Culex* included a long poly-T region with approximately 18 and 9 consecutive Ts, respectively (an example from *An. gambiae* is shown Fig. 2b). The CSM had a poly-A region immediately before the 5 cytosines, the same or similar length as the poly-T region. In KT852976 (*Culex tritaeniorhynchus*) complementary A → T transversions were at the same distance from the boundary of the central poly-C region (Fig. 3). The AnSM had an E-value of 8.3 × 10^–2327^ while the CSM had an E-value of 8.6 × 10^–248^. The *Anopheles* and *Culex* long motifs (AnLM and CLM) had an E-value of 6.5 × 10^–3302^ and 1.3 × 10^–445^ respectively. This suggests that these motifs are highly unlikely to be coincidental. A Short motif (AeSM) was also detected in *Aedes*. In contrast to the short motifs identified in *Anopheles* and *Culex*, the poly-T region in AeSM is less conserved and contains more adenosine residues. Due to the small number of *Aedes* sequences, however, we cannot rule out if this departure from the other short motifs is real.

**Table 3:** Conserved motifs found in mosquito mitogenomes control regions.

Motif	Presence in genus	Motif start site (bp) relative to the end of the 12S rRNA gene	Length	Sequence	E-value
Anopheles short motif (AnSM)	390/393	133–206	25	5’-CCCCT AWTTT TTTTT TTTTT TTTWT-3’	8.3e−2327
Anopheles long motif (AnLM)	388/393	88–162	41	5’-ATWWW TAWTT AATAA ATWWT TWWAG TACAA TTCTC CTTWT A-3’	6.5e−3302
Culex short (CSM)	34/37	129–148	25	5’-AAAAA AMCCC CMATT TTTTT TTGTA-3’	8.3e−248
Culex long (CLM)	34/37	76–95	50	5’-TATMA ATTAT TAAAT WAGAA TWAAW AATAG TATAT TCCTC CCCAA AAYTC-3’	1.3e−445
Aedes short (AeSM)	14/16	130–205	15	5’-CCCTT AAWTW WWTTT-3’	5.4e−38

A small number of atypical sequences in the dataset were observed. Four of 392 *Anopheles* mitogenome sequences, which belong to *An. parvus* (MF381635, MF381645, MF381670) and *An. kompi* (MF381721). In the *An. parvus* sequences, the AnSM and AnLM were located 80 bp farther upstream of the 12S rRNA gene than in other *Anopheles* mitogenome sequences. In MF381721 (*An. kompi*), the AnSM was shifted 80 bp farther upstream, with an additional CLM between the AnLM and AnSM. The final arrangement of motifs was AnLM, CLM, AnSM. Long and short motifs in these *Anopheles* mitogenomes were located 162 bp and 205–206 bp upstream of the 12S rRNA, respectively, and upon closer inspection, the locations of these motifs (relative to the 12S rRNA gene) seem to be correct and there are no homopolymers or short repeat segments observed which could cause sequencing errors.

### The Culex short motif is predicted to form a double-stranded hairpin

The symmetry of the CSM is suggestive of a double-stranded hairpin. To investigate whether the formation of a double-stranded hairpin is possible, the secondary structures of the poly-T and poly-A regions and 15 of the nucleotides on each side were predicted at 5 °C increments from 10 to 50 °C, inclusive. In all 27 *Culex* sequences tested and at all temperatures, the CSM forms a predicted hairpin loop in the stem involving the poly-A and poly-T regions (Fig. 5). Temperature changes only affected the folding of the sequence’s free ends, and the hairpin loop was present in folding simulations up to 50 °C. The hairpin loop and step formed even when the entire CR was used. Similar hairpin structures were not observed with *Anopheles* or *Aedes* sequences.

**Figure 4:**
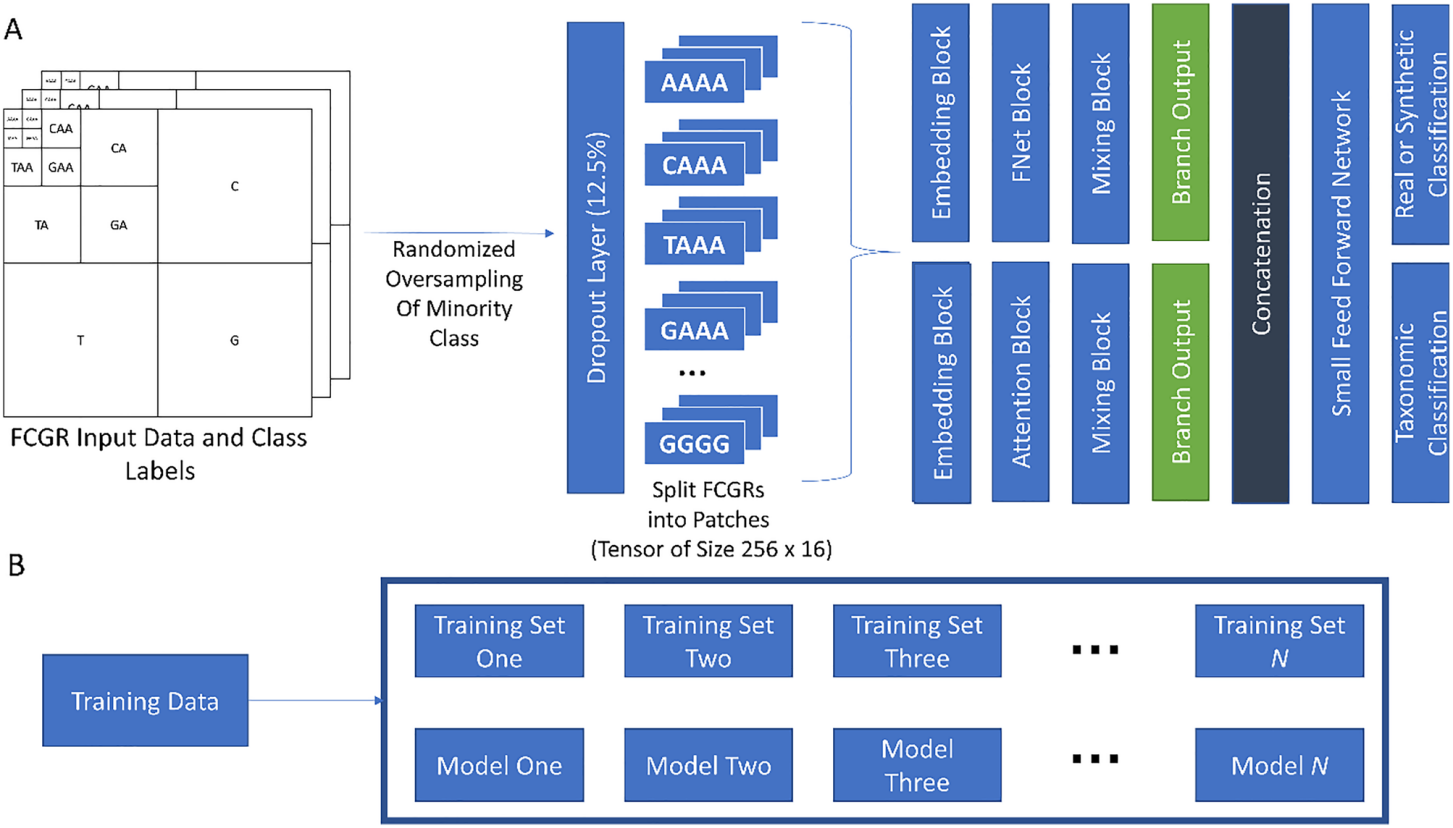
Overview of the deep neural network used to classify mosquito and non-mosquito sequences. (**A**) A simple overview of how information from normalized FCGRs passes through each branch of the network. Each branch begins with FCGRs being split into patches. The information from each patch then passes through attention layers and a small fully connected feed-forward network. The layers predicting target information (real/synthetic, genera) are the last layers of the network. During training, the loss between predictions and actual targets is minimized by gradually adjusting the weights and biases of each layer. When predicting unknown labels, the layer which classifies each FCGR as either real or synthetic is discarded and only the taxonomic classification layer is used. Colors have been added only to aid in visualization. (**B**) The meta-classifier creates random training sets using the training data. The weights of each model are initialized randomly. This helps train a diverse set of models which can be used to classify unseen data.

**Figure 5:**
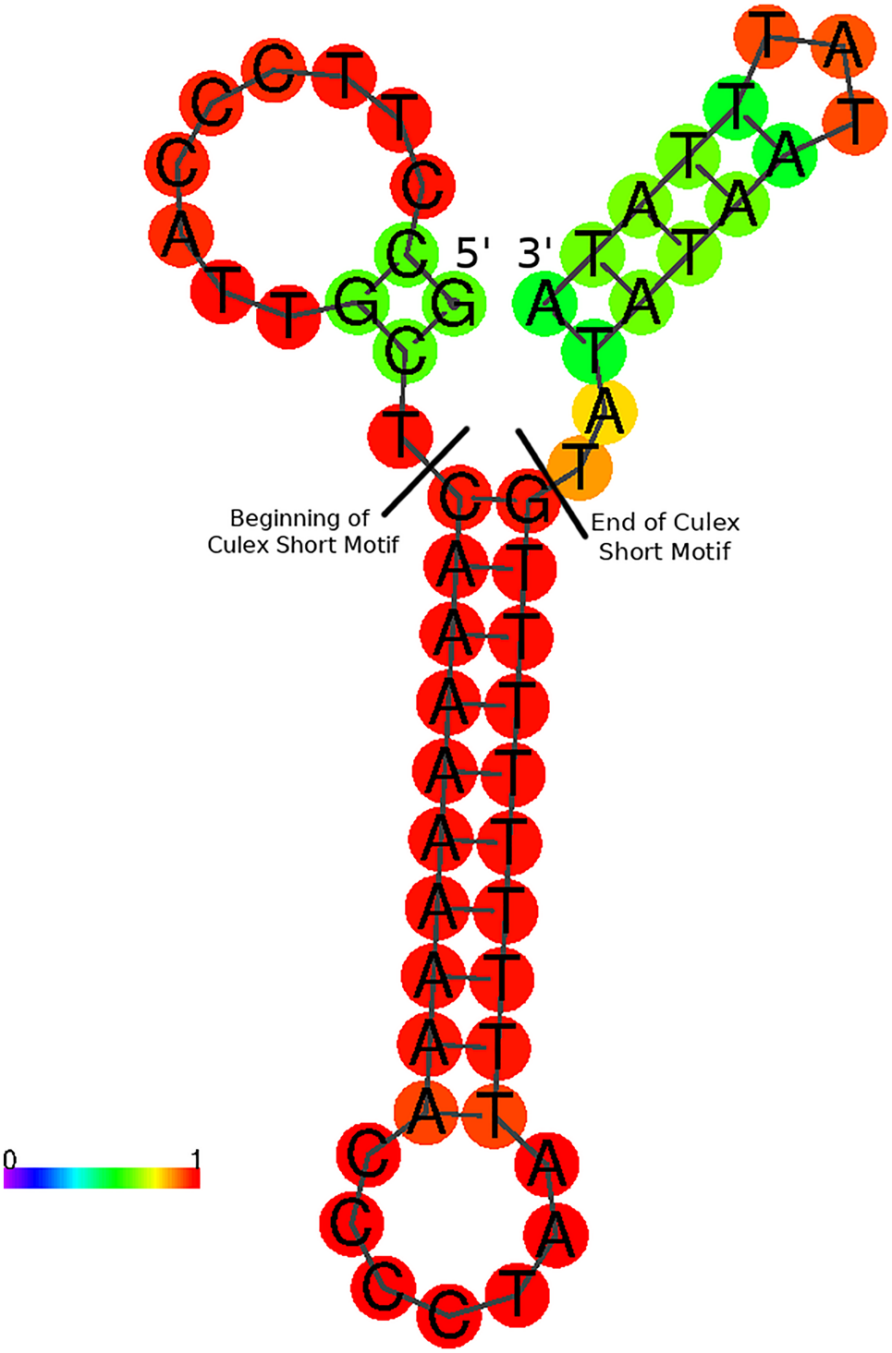
Predicted secondary structure of NC_014574 (*Culex quinquefasciatus*) Culex Short motif and surrounding bases at 25 °C. Coloured scale represents the probability of bases being in the state represented. Bases are coloured according to the likelihood they are in their shown state. Blue/purple bases are unlikely, cyan/green are somewhat likely, and yellow/red are the most likely. Predictions made with ViennaRNA v 2.4.14 using DNA stacking energies.

### CR motifs in Culicidae mitogenomes tend to be conserved within specific genera

The presence of various long and short motifs identified in this study are strongly associated with specific mosquito genera and with the Culicidae in general. This is especially true when the location in the CR and the motif pairs are considered. However, the correlation is not perfect. Our investigation into the genus specificity of the long and short *Anopheles* and *Culex* motifs detected the presence of at least one short or long motif in 25 of 304 (8.2%) non-Culicidae Dipteran (nCD) mitogenome CRs (Table 5). In these organisms, the motifs were found 83–210 bp upstream of the 12S rRNA gene. The presence of these putative motifs in other parts of the nCD CRs was also examined and we determined that 1 nCD sequence (MH321208) had both a long and short *Anopheles* motif present in the correct order on the same strand starting within 128 bp of each other.

While the long and short motifs identified here do appear to be associated with specific mosquito genera (Table 3), rare exceptions were observed (Table 4). For example, 10 Anopheles and Culex CR sequences did not contain a long and/or short motif associated with its genus. Three of these sequences, MK575477 (*An.* cruzii), MK575478 (*An.* darlingi), and MK575480 (*Cx.* quinquefasciatus), were significantly shorter (< 250 bp) than other CRs, suggesting sequencing errors. Four of the sequences, MN389458 (*Cx.* fergusoni), JX219731 (*An.* dirus), MF381720 (*Cx.* chidesteri), and MF381630 (*An.* gilesi), had a Long and Short motif, but one or both not matching the sequence’s genus. Two of the sequences, MF381737 (*An.* pseudotibiamaculatus) and MN389457 (*Cx.* cylindricus), had only one motif, which matched the genus of the sequence. Finally, in MF381721 (*An.* kompi), we observed the CLM and AnLM 160 and 210 bp upstream of the 12S rRNA and a second AnLM was located 97 bp upstream of the 12S rRNA. Three sequences belonging to *An.* parvus (MF381635, MF381670, and MF381645) did not contain motifs in the expected location. In *An.* parvus, the AnLM and AnSM were found 162 bp and 205–206 bp upstream of the 12S rRNA gene, respectively (Table 5).

**Table 4:** Control Region sequences which were annotated with motifs from different genera, or incompletely^a^.

Accession	Species	Expected	Annotated
MF194022, OM214531	Aedes aegypti	Aedes short	None^b^
MK575477	Anopheles cruzii	Anopheles (Short, Long)	None^b^
MK575478	Anopheles darlingi	Anopheles (Short, Long)	None^b^
JX219731	Anopheles dirus	Anopheles (Short, Long)	Anopheles long, Aedes short^b^
MF381630	Anopheles gilesi	Anopheles (Short, Long)	Culex long, Anopheles short
MF381721	Anopheles kompi	Anopheles (Short, Long)	Anopheles long
MF381737	Anopheles pseudotibiamaculatus	Anopheles (Short, Long)	Anopheles short
MF381720	Culex chidesteri	Culex (Short, Long)	Anopheles long, Culex short
MN389457	Culex cylindricus	Culex (Short, Long)	Culex long
MN389458	Culex fergusoni	Culex (Short, Long)	Anopheles long
MK575480	Culex quinquefasciatus	Culex (Short, Long)	None^b^
MF381612	Bironella hollandi	None	Anopheles (Long, Short)
MK575479	Coquillettidia chrysonotum	None	Aedes short
MH316118	Lutzia fuscana	None	Culex long, Aedes short
MH316119	Lutzia halifaxii	None	Culex long, Aedes short
MN342085	Mansonia uniformis	None	Culex (Long, Short)
MK575476	Ochlerotatus fulvus	None	Aedes short
MN389467	Ochlerotatus nigrithorax	None	Anopheles long, Aedes short
MN626442	Ochlerotatus taeniorhynchus	None	Culex long, Aedes short
KP721463	Ochlerotatus vigilax	None	Culex long, Aedes short
MK575484	Ochlerotatus vigilax	None	Aedes short
MN389473	Ochlerotatus vittiger	None	Aedes short
OK662581	Psorophora albipes	None	Aedes short
MF957171	Sabethes belisarioi	None	Culex long, Anopheles short
MN389468	Tripteroides tasmaniensis	None	Anopheles (Long, Short)
MK575492	Wyeomyia confusa	None	Aedes short

^a^Expected motifs are the motifs expected to be found in the region 80–210 bp upstream of the 12S rRNA gene, and Annotated motifs are the motifs annotated by MAST in that region.

^b^Other complete mitogenome sequences from the same species had the expected GSMs.

This table includes 27 of the 472 (5.7%) sequences examined.

**Table 5:** Non-Culicidae Dipteran sequences with the conserved mosquito mitogenome control region motifs in the mitogenome control region between 80 and 210 bp upstream of the 12S rRNA gene.

Motif(s)*	Accession(s)	Species
Anopheles long	MH321208	Eristalinus aeneus
	MG735216	Graphomya rufitibia
	MG252777	Liriomyza chinensis
	GU327644	Liriomyza trifolii
	JN570506	Liriomyza trifolii
	JX913758	Lucilia porphyrina
	KM200723	Musca domestica
	MG941012	Musca sorbens
	MH521132	Musca sorbens
	KM676394	Muscina stabulans
	KR349298	Phlebotomus papatasi
	KT444442	Phlebotomus papatasi
	MH540745	Sarcophaga dux
Anopheles short	JN861747	Cramptonomyia spenceri
	MH321205	Eristalinus barclayi
	MH321204	Eristalinus fuscicornis
	MH159199	Eristalis tenax
	KY679159	Hermetia illucens
	MH705623	Hydrotaea spinigera
	MF434829	Neoceratitis asiatica
Culex long	AJ242872	Ceratitis capitata
	KF824877	Drosophila yakuba
Aedes short	JN861744	Ptychoptera sp. ATB-2011
Anopheles long, Culex short	JN861749	Chironomus tepperi

*Motifs were counted if MAST found the motif on the majority strand, and the starting point was between 83 and 165 bp (for Long motifs) or 130–210 bp (for Short motifs) upstream of the end of the 12S rRNA gene.

In other genera, such as Aedes, one or both expected motifs were missing or not observed in their expected place upstream of the 12S rRNA gene of a small number of sequences. For example, two of four *Ae.* (Stegomyia) aegypti sequences, MF194022 and OM214531, did not contain any motifs in the region 80–200 bp upstream of the 12S rRNA gene and were the only sequences in the Aedes dataset (n = 16) that did not contain any Aedes associated motifs.

### The control region contains sufficient information to classify Culicidae and non-Culicidae dipterans

**Figure 6:**
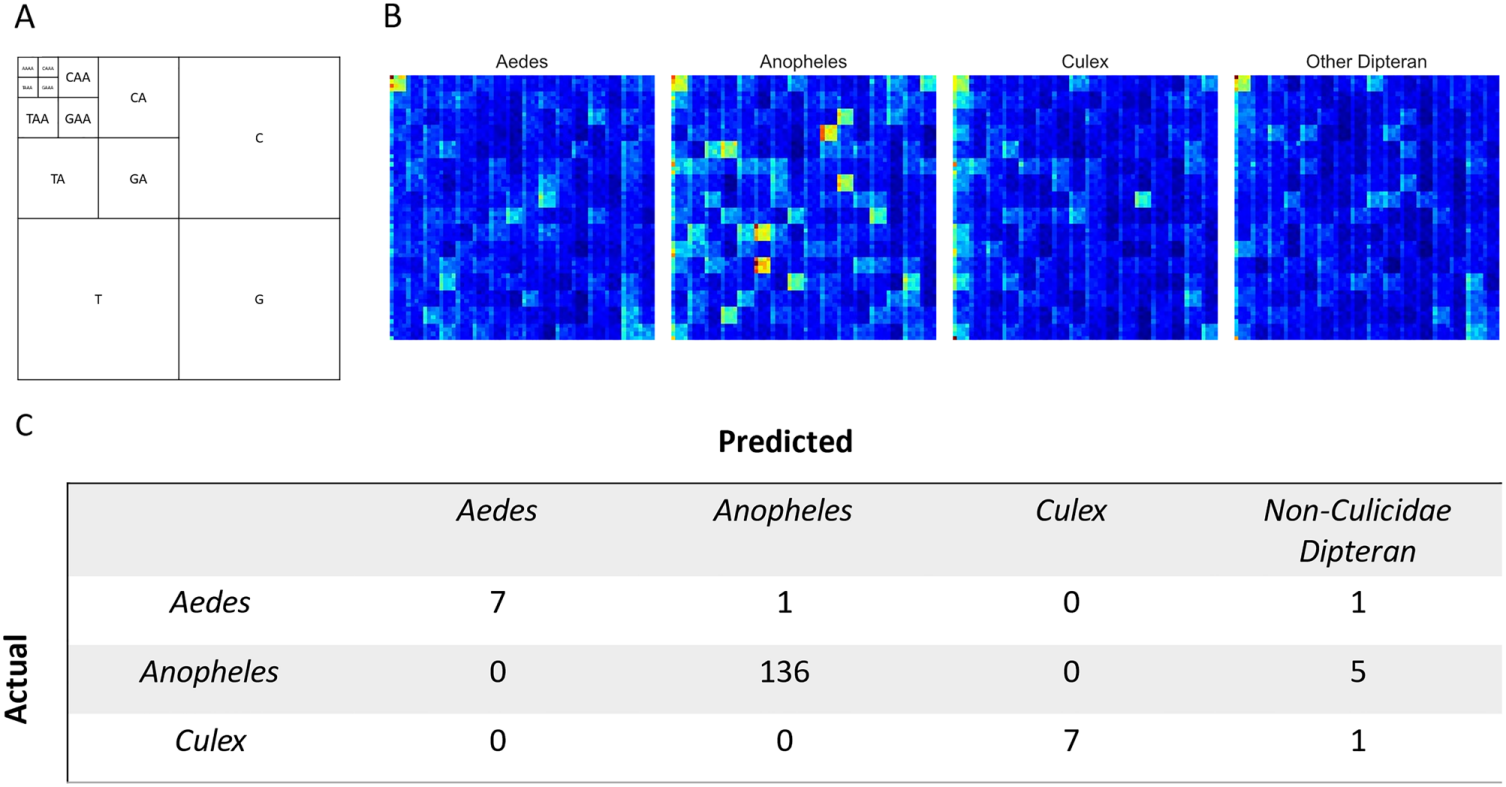
Results of the semi-supervised learning investigation using a deep learning model. The model was trained using data collected up to 2019. (**A**) The organizational structure of the chaos game. An example of the composition of the NAAA super-pixel can be found in the top left corner. (**B**) Saliency map for each FCGR. Highlighted are 4 × 4 super-pixels of k-mer frequencies corresponding to the different regions of the FCGR presented in (**A**). For example, the patch found in the top left corner represents a collection of k-mers ending in AAAA. High saliency regions are warmer and are used by the model to differentiate between sequences. (**C**) This table displays the results of the fivefold stratified cross-validation experiment. Predictions which were correctly made are found where both the column and row labels are identical. False negative predictions for each genus are found along the rows (eg: five Anopheles sequences were predicted to be Non-Culicidae dipterans) while false positives are found along the columns.

**Figure 7:**
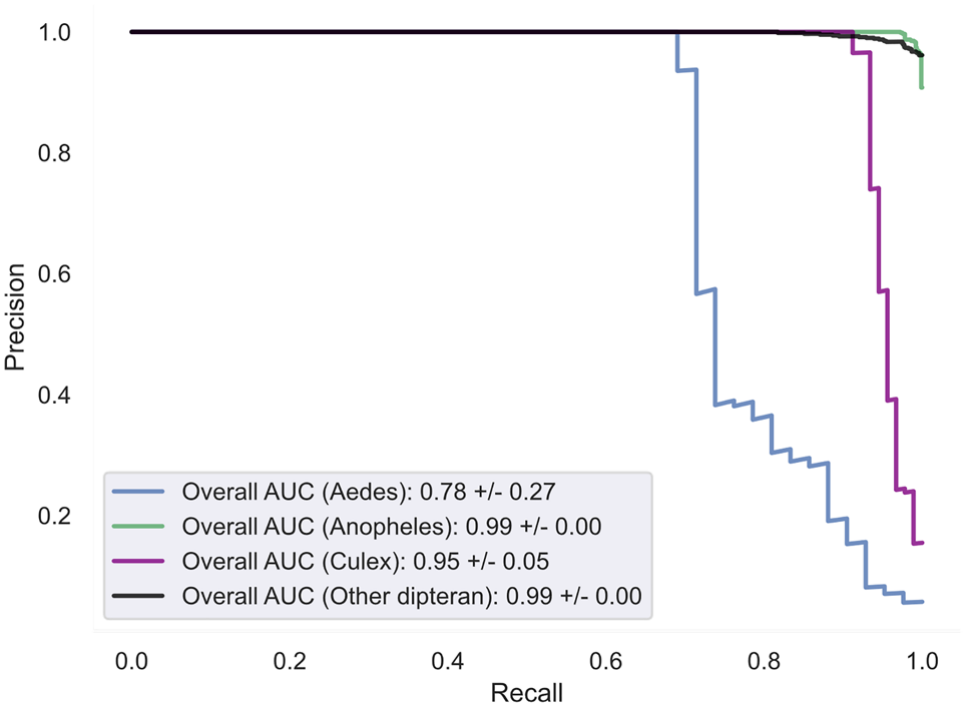
Precision-Recall curve quantifying the performance of the semi-supervised classifier when trained on the entire dataset. Precision-Recall curves illustrate the ability of our deep learning model to balance the identification of true positive sequences while minimizing the number of false identifications (precision) and false negatives (recall).

The supervised learning analysis demonstrated that FCGR representations of the CR could differentiate various Culicidae and non-Culicidae dipterans with high accuracy (Figs. 6 and 7). Unfortunately, genera that are not well represented in this dataset—such as those belonging to the *Aedes*—were more difficult to classify. Saliency maps revealed that frequency in occurrence of specific regions within each FCGR is responsible for differentiating these groups. In *Anopheles spp*., for example, the model used regions of the corresponding to the frequency of *k*-mers ending in AAAA to discriminate between different taxa (Fig. 6B, top left corner of each saliency map). In addition, *k*-mers ending in CCCC, ACTA, CTCA, GTAC, AGAC, AGTC, GTCT were considered when classifying *Anopheles* from other taxa (Fig. 6A and B). Finally, the classifier appeared to be robust to differences in sampling year and made few classification errors (Fig. 7). Finally, although a comparison between alternate learning models was not the goal of this project, the comparisons we conducted demonstrated that both self-supervised deep learning and classical classifiers and their self-supervised versions perform competitively (Fig. 7 and Suppl Fig. 6).

## Discussion

The control region (CR) of mosquitoes are understudied, yet it maybe information rich as it is highly diverse. A total of 472 publicly available mosquito mitogenome CRs were compared in this study. Several genera were not included in the MEME analysis since very few sequences from these genera were present in our database. For example, seven genera have only one sequence from one species present. Given that our results for *Aedes,* using only 16 sequences, did not result in a highly specific motif, the exclusion of genera with single digit sequences was warranted as it may have led to erroneous or uninformative results. General features of the composition of the mitogenomes, DNA sequence motifs in the CR that have the potential to facilitate differentiation of the *Aedes*, *Anopheles*, and *Culex* genera and complement other established DNA barcodes are presented. Pairs of putative motifs, denoted short and long, were identified 80–210 bp upstream of the mosquito mitogenome’s 12S rRNA gene. The conservation of these features in non-coding regions suggests a possible functional role in the mitogenome, though its exact purpose remains to be determined. The extremely low E-values of the motifs give a low likelihood that the motifs found are coincidental artifacts of random noise. With very few exceptions, the motifs we identified were correlated with three mosquito genera. Only five out of the 445 publicly available *Aedes*, *Anopheles*, and *Culex* mitogenome sequences analyzed do not contain any of the motifs identified in this study in the region 80–200 bp upstream of the 12S rRNA. Of these, two belong to *Ae. aegypti*, and one each of *An. cruzii*, *An. darlingi*, and *Cx. quinquefasciatus*. These species all have other publicly available sequences that were analyzed in this study with the correct motifs for the genus.

The short motif appears to have analogs in other mosquito and insect mitogenomes and there is evidence that suggests that the poly-T stretch of the short motif has a possible functional role in the signaling of the origin of replication for the minor strand ^15, 19, 21, 52^. Similar poly-T stretches to that of the AnSM have been observed in the mitogenome CRs of other insects, such as *Bombyx mori*, *Tribolium castaneum*, *Locusta migratoria*, and *Drosophila* spp. ^19^. However, these occur at different locations than those reported here^19^. In *Drosophila* spp., for example, a poly-T stretch was found 437–511 bp upstream from the tRNA^Ile^ gene while stretches in *Tribolium castaneum* and *Locusta migratoria migratoria* are found 657–683 bp and 576–577 bp upstream from the tRNA^Ile^ gene, respectively. In *Bombyx mori* this region is located 470–471 bp upstream from the tRNA^Met^ gene^15^. Our work has found structurally similar poly-T stretches in all 389 *Anopheles* CR sequences and in 36 of the 37 *Culex* CR sequences, with MK575480 (*Cx. quinquefasciatus*) being the only outlier. Since this was the only available mitogenome sequence for this species, additional targeted sequencing of the CR is needed to determine if this is an error in the public data and to resolve any ambiguities.

In addition to being well-conserved within their clades, motifs associated with *Anopheles* and *Culex* are rarely present in other non-Culicidae Diptera (nCD) species, especially when considering the expected pairing, order, and location of these motifs within the CR. This work further supports a growing body of evidence that motifs within the CR of mosquito mitogenomes are conserved within the Culicidae and potentially within the sub-families of the Culicidae. Of the 304 nCD mitogenomes analyzed only JN861749 (*Chironomus tepperi*) contained a pair of short and long motifs in the expected region (80–200 bp upstream of the 12S rRNA gene). These motifs were associated with *Culex* and *Anopheles*, respectively. 23 of the 304 nCD CR sequences (7.6%) had one motif present from any mosquito genera in the expected region.

Although we found putative *Aedes* specific motifs, the quality of these motifs tended to be lower since they were the shortest and had lower statistical significance than their *Anopheles* and *Culex* counterparts. This is likely due to the small number of *Aedes* sequences in the data set. Only 16 *Aedes* mitogenome sequences from eight species were publicly available for this study. Additional sequences from a greater number of *Aedes* species will be required to identify high-confidence motifs.

The CSM, which contains an adenine stretch followed by a run of 4–5 cytosine residues and a thymine stretch, may form a hairpin structure. The near-perfect complement of adenine to thymine in and near CSMs suggests biological importance. Similar stem-loop structures have been identified in the CR of other insects, including one in peltoperlid stoneflies (Plecoptera: Peltoperlidae), which was centered around a GGGGGC sequence^18^. MF381722 (*Cx. bilineatus*) and MF381718 (*Cx. lygrus*) do not have perfect length complementarity between the poly-A and poly-T regions, however, hairpin structures are predicted to form at all temperatures up to 50 °C tested. Interestingly, the poly-A stretch was only present in Culex CSM sequences and not present in any other motif. MF381722 (*Cx. bilineatus*) and MF381718 (*Cx. lygrus*) have slightly different lengths of poly-A and poly-T regions with 9 A–7 T, and 10 A–9 T residues, respectively. However, this may be due to errors resulting from sequencing homopolymeric regions. In MF381718 (*Cx. lygrus*) a guanine residue is present in the poly-A stretch. The presence of this residue is predicted to form a bulge in the hairpin. While the poly-A stretch in CSM appeared significant, additional work is needed to better understand the biological significance of this motif.

Mosquito CR sequences in genera not belonging to *Anopheles*, *Culex*, or *Aedes* were mostly unlabelled or labelled with mismatched motifs (such as CLM and AnSM), and only rarely labelled with the motifs of the genus most closely related. Depending on the gene analyzed, *Bironella* is generally placed as a sister genus to *Anopheles*^53^, though in some cases it has been placed within *Anopheles*^54^. Thus, the annotation of *Bironella* sequences in the study with the *Anopheles* GSMs is consistent with the current mosquito phylogeny^55^. However, four sequences were unexpectedly annotated with GSMs from more distant genera. *Mansonia* is most closely related to Aedes, yet MN342085 (*Mansonia uniformis*) was annotated with CLM and CSM. *Tripteroides*, *Wyeomyi*a, and *Sabethes* spp. (part of the Sabethini tribe) are more closely related to *Culex* and *Aedes* than *Anopheles*^56^, but the CLM and AnSM appeared in MF957171 (*Sa. belisarioi*), AnLM and AnSM in MN389468 (*Tripteroides tasmaniensis*), and MK575492 (*Wyeomyia confusa*) was annotated with the AeSM. These sequences being annotated with the CLM and CSM would be more in line with the current understanding of the mosquito phylogeny. The other three *Sabethes* spp. sequences in the dataset were not annotated with any motifs described for the three genera.

While the motifs identified in this work appear to be unique to each mosquito genera, the relatively short length and low complexity of these motifs could prevent this region from being a universal barcode unique to Culicidae. However, if one considers that these motifs are embedded in a broader context, the entire CR, a machine learning approach may be useful in delineating closely related taxonomic groups or species complexes^12, 18, 57, 58^. FCGRs could also be useful in understanding broad patterns in nucleotide usage and k-mer usage in the CR and in detecting palindromes and tandem repeats^26, 33^. FCGRs are also useful since the genus or species signature should still be present even in the presence of ambiguous nucleotides^25, 59^. Given this, we believe our choice in using FCGRs for classification is warranted because, at a minimum, they have been shown to be useful in coarse-grained classification and motif identification tasks^26, 33, 35, 59^. Our experiments support this hypothesis and demonstrate that the FCGR of the CR is informative, and supervised and semi-supervised approaches can result in highly predictive models (Figs. 6 and 7, Suppl Fig. 6). The deep learning model presented in our work is a proof-of-concept demonstrating the potential and flexibility that machine learning using genomic signatures can provide when performing taxonomic assignments. These models can be trained using a limited number of training samples to produce an accurate discriminatory model^60^. Semi-supervised approaches, such as SGANs, are particularly useful in this domain since obtaining additional training data can be a time-consuming and expensive task^60^. The performance of our deep model is particularly impressive since the supervised discriminator of each individual model in the deep learning ensemble was trained on approximately 9.5% of the training data (64 out of 673 total samples). In comparison, 60 and 100% of the training data was used to train each of the self-supervised and fully-supervised models. Also, although our model performed well, a much larger comparative study investigating the advantages and suitability of deep-learning and signature-based approaches have over other taxonomic classifiers, such as the RDP Classifier, needs to be undertaken^24^. This is necessary since some algorithms may be better at identifying samples at higher taxonomic ranks while failing to differentiate between closely related species due to the lack of training examples. In areas where identification is important, such as identifying the presence of disease vectors, an elevated false-negative or positive rate is unacceptable. We believe that the approach presented here and others (such as one-shot and few-shot learning) have significant potential for being used as a basis for classifying reads from environmental samples since genomic signatures and deep-neural networks can capture global patterns within genomes that other approaches may miss^29, 34, 61^. For example, FCGRs can be seen as more than just *k*-mer counts since visual information about the overall structure of sequence, along with *k*-mer counts, is encoded into the representation^29, 34, 62^. When this information is processed by a deep neural network, such as the SGAN employed here, the hidden layers of the discriminator network likely learn a meaningful and distilled representation of the FCGR signature which enables accurate classification^26–28^.

Additional work needs to be conducted into ways which may improve the quality of genomic signatures and models trained using these signatures. Long-read sequencing technologies can be particularly beneficial since considerably more information about each sequence can be extracted from the longer reads. Furthermore, these technologies allow homopolymeric regions, such as the CR, to be sequenced in their entirety. Recently, we have developed PCR primers for the amplification of mosquito mitochondrial CR and these have been successfully used on 28 Canadian mosquito species (manuscript in preparation). PCR primers for the amplification of the control region will be a useful tool as it will allow accurate sequencing of he region from single, as well as, bulk metagenomic samples. Together, this can improve the quality of the FCGRs for each species which could allow supervised and semi-supervised machine learning models to learn a more robust representation of each species^26, 28, 36^. Furthermore, since the CR is an understudied, the development of these primers and computational methods will allow for a more thorough investigation of the CR and its motifs in future studies of this region. Long-read sequencing also has the added benefit in that it can potentially recover sequences of less well-represented organisms for which adequate primers are not available, resulting in larger and more complete databases which can be used to further refine taxonomic classification models. While not perfect, long read sequencing methods and new primers specifically targeting the CR can begin to help resolve ambiguities arising from homo-polymer tracts, high AT content, and repeating regions found in the CR. While we did perform data cleaning to remove a large number of low quality and incomplete mitochondrial sequences, we cannot be absolutely sure of the accuracy of every sequence in the dataset since information on the sequencing methods used, depth of coverage, and the methods used to assemble each sequence in our study is not available. Additional sequences from species and genera that are poorly represented in currently available sequence databases will be useful in expanding this study. Finally, machine learning models could potentially be used to identify specific sequences within the CR which are useful for delineating species. These sequences could serve as a starting point for new investigations which aim to determine the biological functions associated with the CR and if this region can be used to study mosquito population genetics.

In conclusion, we performed an in-silico analysis of 472 complete mitogenome sequences from 125 species of mosquito. This analysis discovered highly conserved motifs, two in the mitogenomes from *Anopheles spp*. and two from the mitogenomes of the different *Culex spp.* Also, although small in number, our analysis of the various *Aedes* mitogenomes also suggested the presence of two potentially conserved motifs and that the average length of the CR from *Aedes* were the longest. Encouragingly, the FCGR signature of the CR was able to distinguish between the mosquito lineages with a high degree of accuracy. We suspect that as additional sequences are added the generalization performance of machine learning models using genomic signatures will improve, especially when tasked to classify rare sequences. We have also demonstrated how these models can be used to investigate which aspects of the signature are important for classification, potentially leading to a better understanding of the functions associated with the CR. This work can have implications in the efficient identification of important mosquito genera known to carry human pathogens, particularly from bulk samples.

## Supplementary Information

Supplementary Information.

## Data Availability

The datasets generated analyzed during the current study are available at the following GitHub page: jrudar/In-Silico-Identification-of-Multiple-Conserved-Motifs-Within-the-CR-of-Culicidae-Mitogenomes: Data for the manuscript titled “In-Silico Identification of Multiple Conserved Motifs Within the Control Region of Culicidae Mitogenomes” (https://github.com/jrudar).

## References

[1] Harbach RE, Besansky NJ (2014). Mosquitoes. Curr. Biol..

[2] World Malaria Report 2019. Available from: https://www.who.int/publications-detail-redirect/9789241565721 (2020).

[3] Ruzzante L, Reijnders MJMF, Waterhouse RM (2019). Of genes and genomes: mosquito evolution and diversity. Trends Parasitol..

[4] Lourens GB, Ferrell DK (2019). Lymphatic filariasis. Nurs. Clin. N. Am..

[5] Musso D, Gubler DJ (2016). Zika virus. Clin. Microbiol. Rev..

[6] Rift HA, Fever V (2017). Rift valley fever. Clin. Lab. Med..

[7] Hubálek Z, Rudolf I, Nowotny N, Maramorosch K, Murphy FA (2014). Chapter five—Arboviruses Pathogenic for domestic and wild animals. Advances in Virus Research.

[8] Simon, L. V. & Fischer, M. A. Western equine encephalitis. In *StatPearls* (StatPearls Publishing, Treasure Island (FL), 2020). Available from: http://www.ncbi.nlm.nih.gov/books/NBK470228/. 29262096

[9] Brugueras S, Fernández-Martínez B, Martínez-de la Puente J, Figuerola J, Porro TM, Rius C (2002). Environmental drivers, climate change and emergent diseases transmitted by mosquitoes and their vectors in southern Europe: A systematic review. Environ. Res..

[10] Ballard JWO, Whitlock MC (2004). The incomplete natural history of mitochondria. Mol. Ecol..

[11] Amorim A, Fernandes T, Taveira N (2019). Mitochondrial DNA in human identification: A review. PeerJ.

[12] Guo J, Yan ZT, Fu WB, Yuan H, Li XD, Chen B (2021). Complete mitogenomes of Anopheles peditaeniatus and Anopheles nitidus and phylogenetic relationships within the genus Anopheles inferred from mitogenomes. Parasites Vectors.

[13] Cameron SL (2014). Insect mitochondrial genomics: Implications for evolution and phylogeny. Annu. Rev. Entomol..

[14] Jourdain F, Picard M, Sulesco T, Haddad N, Harrat Z, Sawalha SS (2018). Identification of mosquitoes (Diptera: Culicidae): An external quality assessment of medical entomology laboratories in the MediLabSecure Network. Parasites Vectors.

[15] Saito S, Tamura K, Aotsuka T (2005). Replication origin of mitochondrial DNA in insects. Genetics.

[16] Zhang DX, Hewitt GM (1997). Insect mitochondrial control region: A review of its structure, evolution and usefulness in evolutionary studies. Biochem. Syst. Ecol..

[17] Demari-Silva B, Foster PG, de Oliveira TMP, Bergo ES, Sanabani SS, Pessôa R (2015). Mitochondrial genomes and comparative analyses of *Culex camposi*, *Culex coronator*, *Culex usquatus* and *Culex usquatissimus* (Diptera:Culicidae), members of the coronator group. BMC Genom..

[18] Schultheis AS, Weigt LA, Hendricks AC (2002). Arrangement and structural conservation of the mitochondrial control region of two species of Plecoptera: Utility of tandem repeat-containing regions in studies of population genetics and evolutionary history. Insect Mol. Biol..

[19] Dueñas JCR, Gardenal CN, Llinás GA, Panzetta-Dutari GM (2006). Structural organization of the mitochondrial DNA control region in Aedes aegypti. Genome.

[20] Caccone A, Garcia BA, Powell JR (1996). Evolution of the mitochondrial DNA control region in the Anopheles gambiae complex. Insect Mol. Biol..

[21] Krzywinski J, Li C, Morris M, Conn JE, Lima JB, Povoa MM (2011). Analysis of the evolutionary forces shaping mitochondrial genomes of a Neotropical malaria vector complex. Mol. Phylogenet. Evol..

[22] Beebe NW (2018). DNA barcoding mosquitoes: Advice for potential prospectors. Parasitology.

[23] Hebert PDN, Cywinska A, Ball SL, deWaard JR (2003). Biological identifications through DNA barcodes. Proc. Biol. Sci..

[24] Wang Q, Garrity GM, Tiedje JM, Cole JR (2007). Naive Bayesian classifier for rapid assignment of rRNA sequences into the new bacterial taxonomy. Appl. Environ. Microbiol..

[25] Cartes, J. A., Anand, S., Ciccolella, S., Bonizzoni, P. & Della Vedova, G. Accurate and fast clade assignment via deep learning and frequency chaos game representation. bioRxiv. Available from: https://www.biorxiv.org/content/early/2022/06/13/2022.06.13.495912 (2022). 10.1093/gigascience/giac119 PMC9795481 36576129

[26] Rizzo, R., Fiannaca, A., La Rosa, M. & Urso, A. Classification experiments of DNA sequences by using a deep neural network and chaos game representation (2016).

[27] Odena, A. Semi-supervised learning with generative adversarial networks. arXiv. Available from: https://arxiv.org/abs/1606.01583 (2016).

[28] Camargo G, Bugatti PH, Saito PTM (2020). Active semi-supervised learning for biological data classification. PLoS ONE.

[29] Jeffrey HJ (1990). Chaos game representation of gene structure. Nucleic Acids Res..

[30] Mitchell JK, Hellberg RS (2016). Use of the mitochondrial control region as a potential DNA Mini-barcoding target for the identification of Canned Tuna species. Food Anal. Methods.

[31] Yang L, Tan Z, Wang D, Xue L, Guan MX, Huang T (2014). Species identification through mitochondrial rRNA genetic analysis. Sci. Rep..

[32] Bailey TL, Boden M, Buske FA, Frith M, Grant CE, Clementi L (2009). MEME Suite: Tools for motif discovery and searching. Nucleic Acids Res..

[33] Vinga S, Carvalho AM, Francisco AP, Russo LM, Almeida JS (2012). Pattern matching through Chaos Game Representation: Bridging numerical and discrete data structures for biological sequence analysis. Algorithms Mol. Biol..

[34] Löchel H, Heider D (2021). Chaos Game Representation and its applications in bioinformatics. Comput. Struct. Biotechnol. J..

[35] Hatje K, Kollmar M (2012). A phylogenetic analysis of the Brassicales clade based on an alignment-free sequence comparison method. Front. Plant Sci..

[36] Ni H, Mu H, Qi D (2021). Applying frequency chaos game representation with perceptual image hashing to gene sequence phylogenetic analyses. J. Mol. Graph. Model..

[37] Dosovitskiy, A., Beyer, L., Kolesnikov, A., Weissenborn, D., Zhai, X., Unterthiner, T. *et al.* An Image is worth 16x16 words: Transformers for image recognition at scale. arXiv. Available from: https://arxiv.org/abs/2010.11929 (2020).

[38] Lee-Thorp, J., Ainslie, J., Eckstein, I. & Ontanon, S. FNet: Mixing tokens with Fourier transforms. arXiv. Available from: https://arxiv.org/abs/2105.03824 (2021).

[39] Lemaître G, Nogueira F, Aridas CK (2017). Imbalanced-learn: A python toolbox to tackle the curse of imbalanced datasets in machine learning. J. Mach. Learn. Res..

[40] Abadi, M., Agarwal, A., Barham, P., Brevdo, E., Chen, Z., Citro, C. *et al.* TensorFlow: Large-scale machine learning on heterogeneous systems. arXiv. https://arxiv.org/abs/1603.04467 (2016)

[41] Tolstikhin, I., Houlsby, N., Kolesnikov, A., Beyer, L., Zhai, X., Unterthiner, T. *et al.* MLP-mixer: An all-MLP architecture for vision. arXiv. Available from: https://arxiv.org/abs/2105.01601 (2021).

[42] Misra, D. Mish: A self regularized non-monotonic activation function. arXiv. https://arxiv.org/abs/1908.08681 (2020).

[43] Salimans, T., Goodfellow, I., Zaremba, W., Cheung, V., Radford, A. & Chen, X. Improved techniques for training GANs. arXiv. Available from: https://arxiv.org/abs/1606.03498 (2016).

[44] Srivastava N, Hinton G, Krizhevsky A, Sutskever I, Salakhutdinov R (2014). Dropout: A simple way to prevent neural networks from overfitting. J. Mach. Learn. Res..

[45] Fort, S., Hu, H. & Lakshminarayanan, B. Deep ensembles: A loss landscape perspective (2020).

[46] Loshchilov, I. & Hutter, F. Decoupled weight decay regularization. arXiv. Available from: https://arxiv.org/abs/1711.05101 (2017).

[47] Yong, H., Huang, J., Hua, X. & Zhang, L. Gradient centralization: A new optimization technique for deep neural networks. arXiv. Available from: https://arxiv.org/abs/2004.01461 (2020).

[48] Zhang, M. R., Lucas, J., Hinton, G. & Ba, J. Lookahead Optimizer: k steps forward, 1 step back. arXiv. Available from: https://arxiv.org/abs/1907.08610 (2019).

[49] Pedregosa F, Varoquaux G, Gramfort A, Michel V, Thirion B, Grisel O (2011). Scikit-learn: Machine learning in python. J. Mach. Learn. Res..

[50] Geurts P, Ernst D, Wehenkel L (2006). Extremely randomized trees. Mach. Learn..

[51] Yarowsky, D. Unsupervised word sense disambiguation rivaling supervised methods. In *Proceedings of the 33rd Annual Meeting on Association for Computational* 189–96. USA: Association for Computational Linguistics. (ACL ’95). Available from: 10.3115/981658.981684 (1995).

[52] Beard CB, Hamm DM, Collins FH (1993). The mitochondrial genome of the mosquito Anopheles gambiae: DNA sequence, genome organization, and comparisons with mitochondrial sequences of other insects. Insect Mol. Biol..

[53] Krzywinski J, Wilkerson RC, Besansky NJ (2001). Toward understanding Anophelinae (Diptera, Culicidae) phylogeny: Insights from nuclear single-copy genes and the weight of evidence. Syst. Biol..

[54] Foster PG, de Oliveira TMP, Bergo ES, Conn JE, Sant’Ana DC, Nagaki SS (2017). Phylogeny of Anophelinae using mitochondrial protein coding genes. R. Soc. Open Sci..

[55] da Silva AF, Machado LC, de Paula MB, da Silva Pessoa Vieira CJ, de Morais Bronzoni RV, de Melo Santos MA (2020). Culicidae evolutionary history focusing on the Culicinae subfamily based on mitochondrial phylogenomics. Sci. Rep..

[56] de Aragão AS, Nunes Neto JP, Cruz ACR, Casseb SMM, Cardoso JF, da Silva SP (2019). Description and phylogeny of the mitochondrial genome of Sabethes chloropterus, Sabethes glaucodaemon and Sabethes belisarioi (Diptera: Culicidae). Genomics.

[57] Bronstein O, Kroh A, Haring E (2018). Mind the gap! The mitochondrial control region and its power as a phylogenetic marker in echinoids. BMC Evol. Biol..

[58] Sun L, Li TJ, Fu WB, Yan ZT, Si FL, Zhang YJ (2019). The complete mt genomes of *Lutzia halifaxia*, *Lt. fuscanus* and *Culex pallidothorax* (Diptera: Culicidae) and comparative analysis of 16 *Culex* and *Lutzia* mt genome sequences. Parasites Vectors.

[59] Lichtblau D (2019). Alignment-free genomic sequence comparison using FCGR and signal processing. BMC Bioinform..

[60] Wambugu N, Chen Y, Xiao Z, Tan K, Wei M, Liu X (2021). Hyperspectral image classification on insufficient-sample and feature learning using deep neural networks: A review. Int. J. Appl. Earth Obs. Geoinf..

[61] Lee, S. H., Lee, S. & Song, B. C. Vision transformer for small-size datasets. arXiv. Available from: https://arxiv.org/abs/2112.13492 (2021).

[62] Millán Arias P, Alipour F, Hill KA, Kari L (2022). DeLUCS: Deep learning for unsupervised clustering of DNA sequences. PLoS ONE.

