# Supplementary material for: In silico identification of multiple conserved motifs within the control region of Culicidae mitogenomes
Source: Sci Rep. 2022 Dec 19;12:21920. doi: 10.1038/s41598-022-26236-5 (PMC9763401; doi:10.1038/s41598-022-26236-5)
Supplement: Supplementary file 1 — Supplementary Information. [file 41598_2022_26236_MOESM1_ESM.docx]

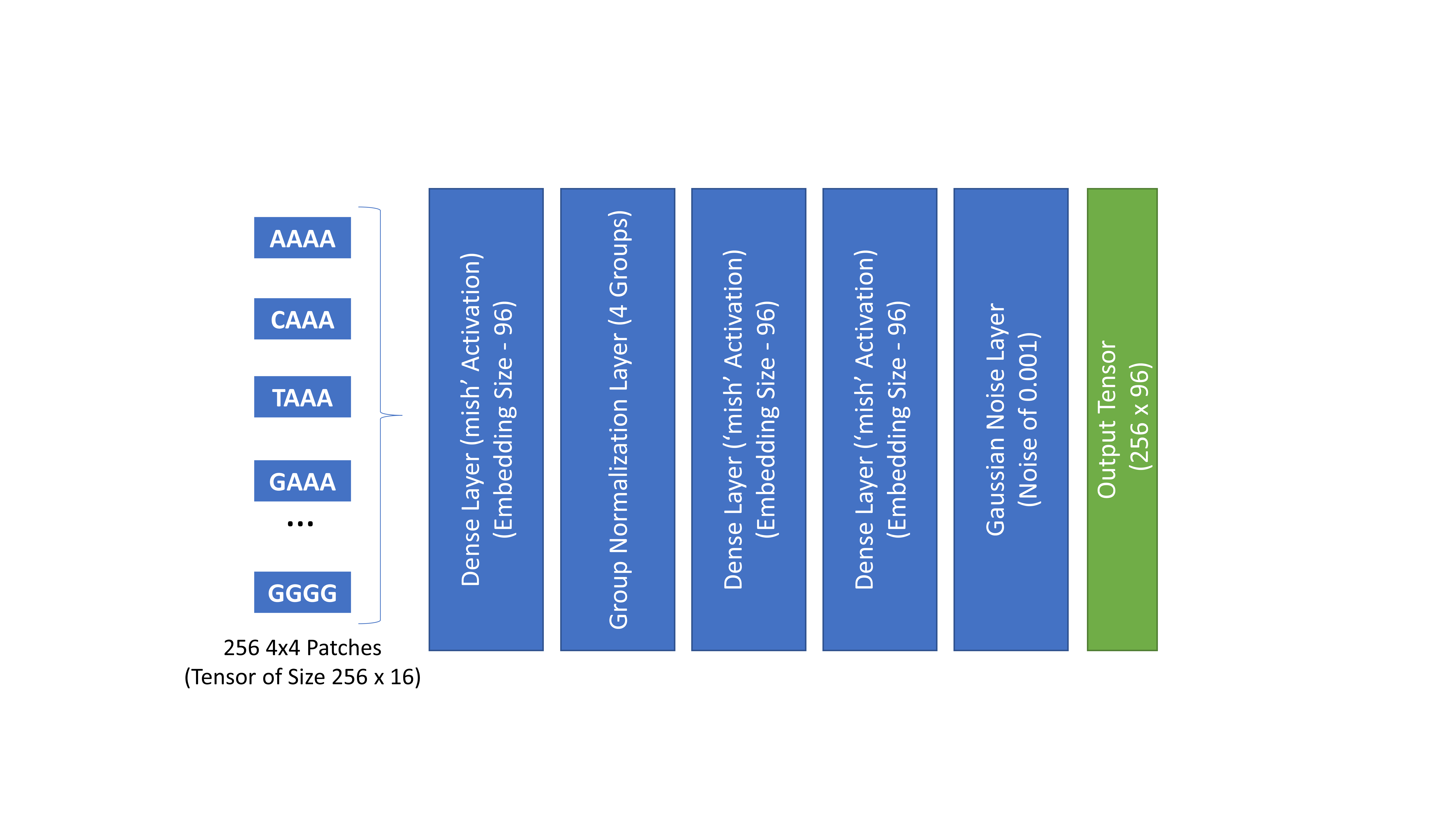


Supplementary Figure 1: Organization of the embedding block. Patches are projected through a series of dense layers before being passed to the next part of the network. A small amount of Gaussian noise is added to the output of the final dense layer to minimize any potential overfitting.


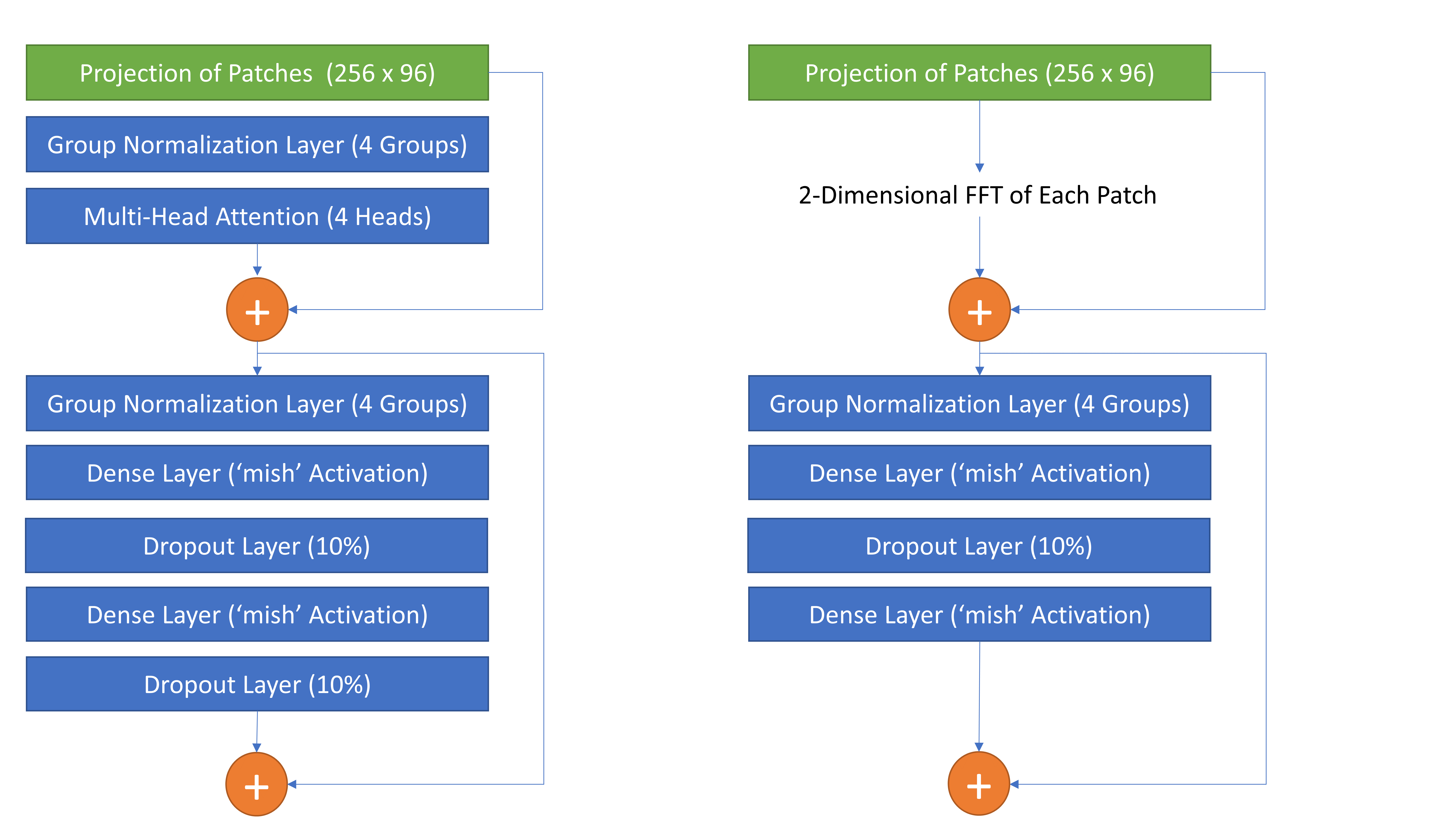


Supplementary Figure 2: Organization of the attention (left) an FNet (right) blocks. Note, within this block the FFT represents the Fast Fourier Transformation.


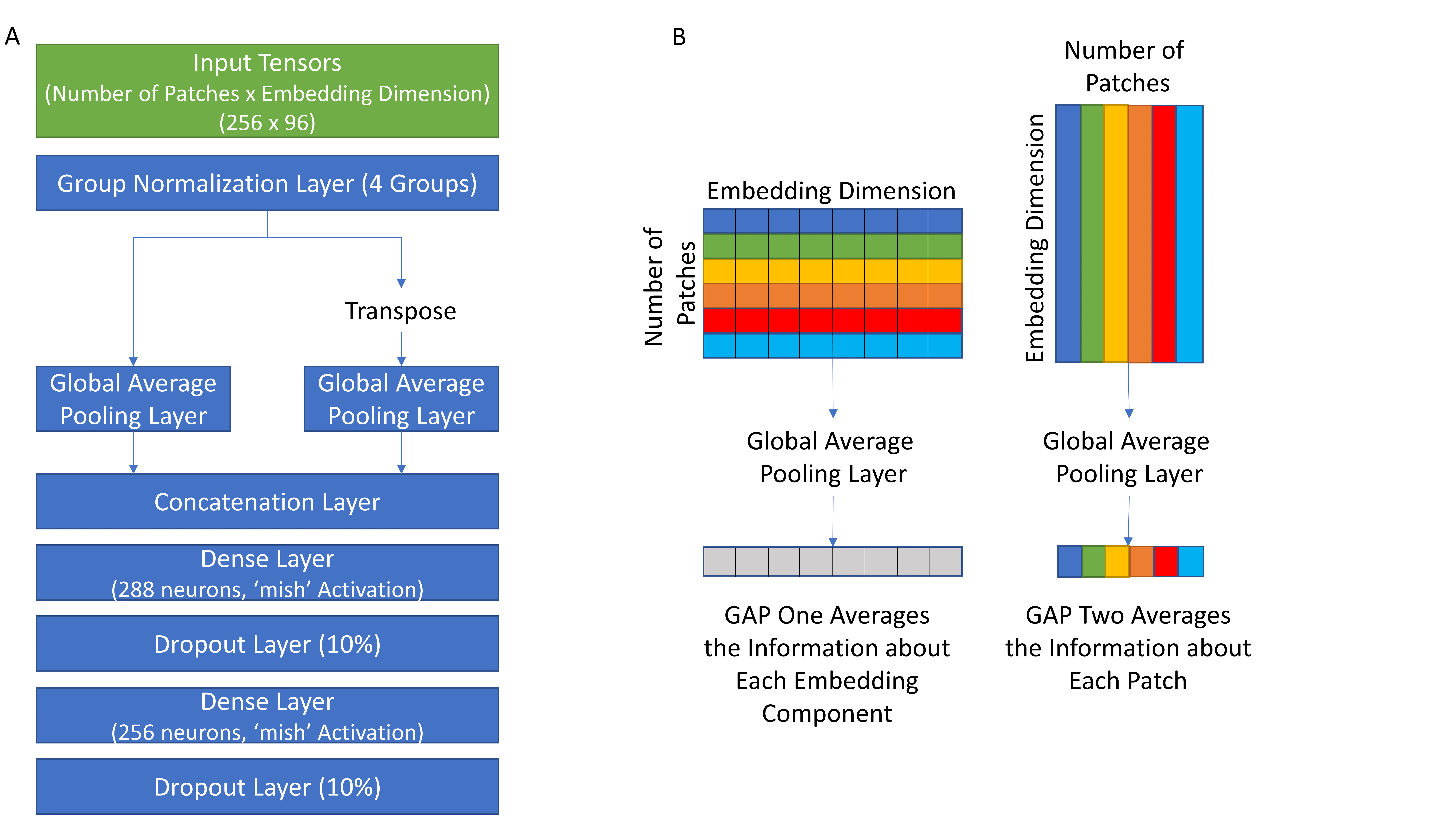


Supplementary Figure 3: Organization of and structure of the mixing blocks. The output of the Attention and FNet blocks are used as input to the Mixing block (A). The mixing block averages the outputs across patches and embedding dimensions (B). The outputs are concatenated and then projected. Dropout is used to prevent overfitting.


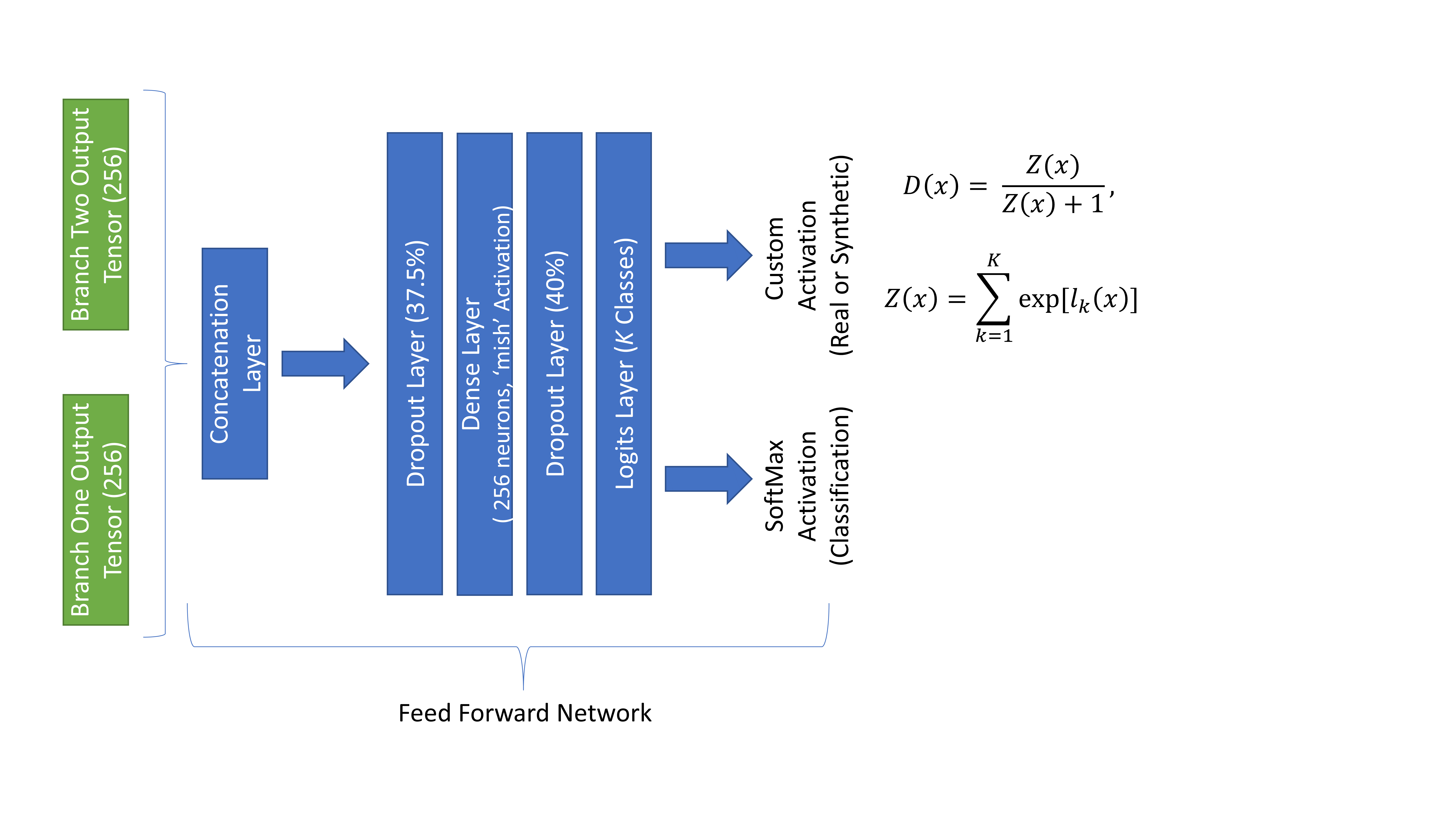


Supplementary Figure 4: Organization of the feed forward network. The output of each branch is concatenated and passed through a feed-forward network for classification. A custom activation function exponentiates then sums the output of the logits layer before normalizing the output by dividing by this sum and adding one.


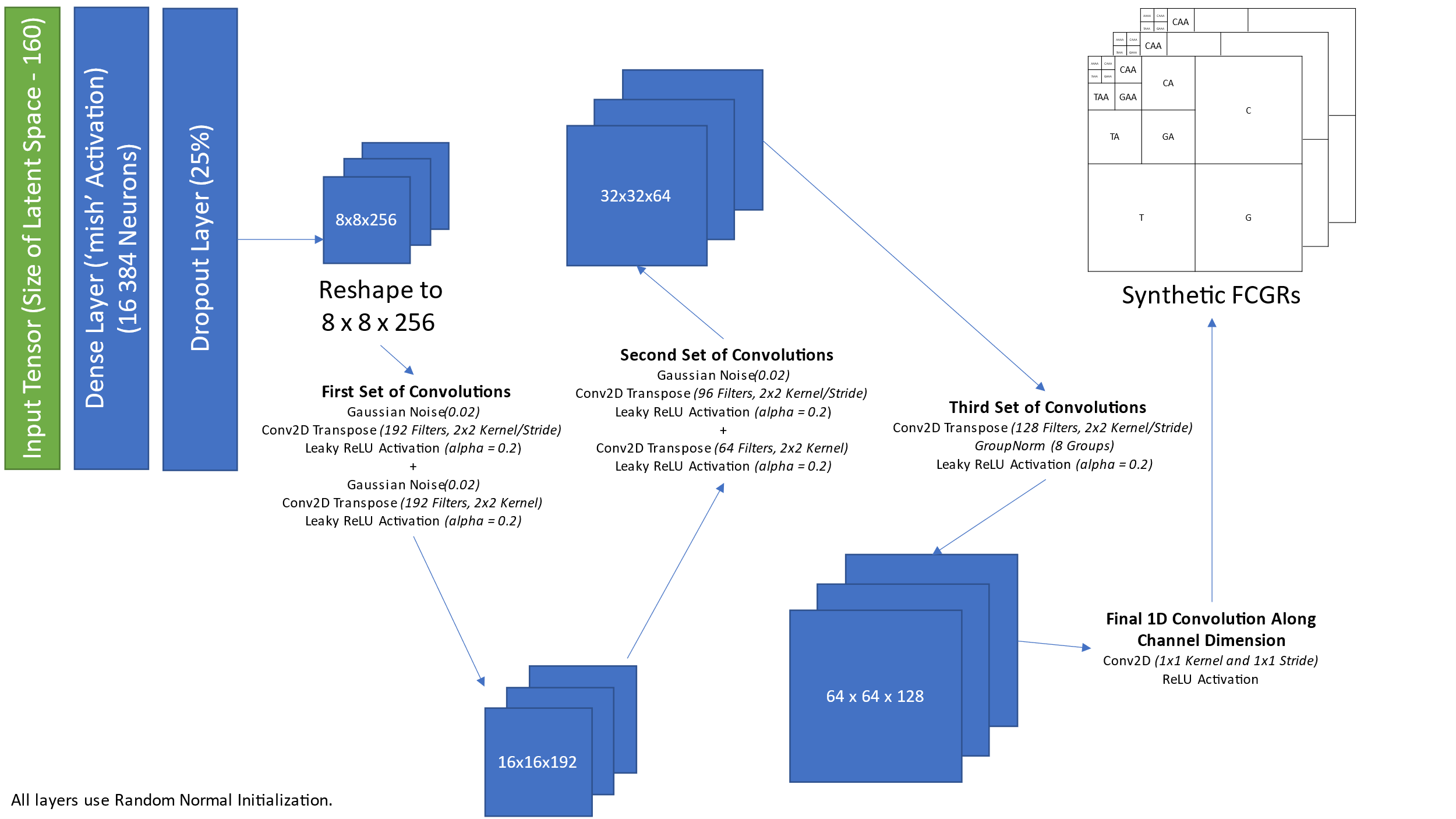


Supplementary Figure 5: Organization of the generative adversarial network. Weights are updated after computing the binary cross-entropy loss of the unsupervised discriminator.


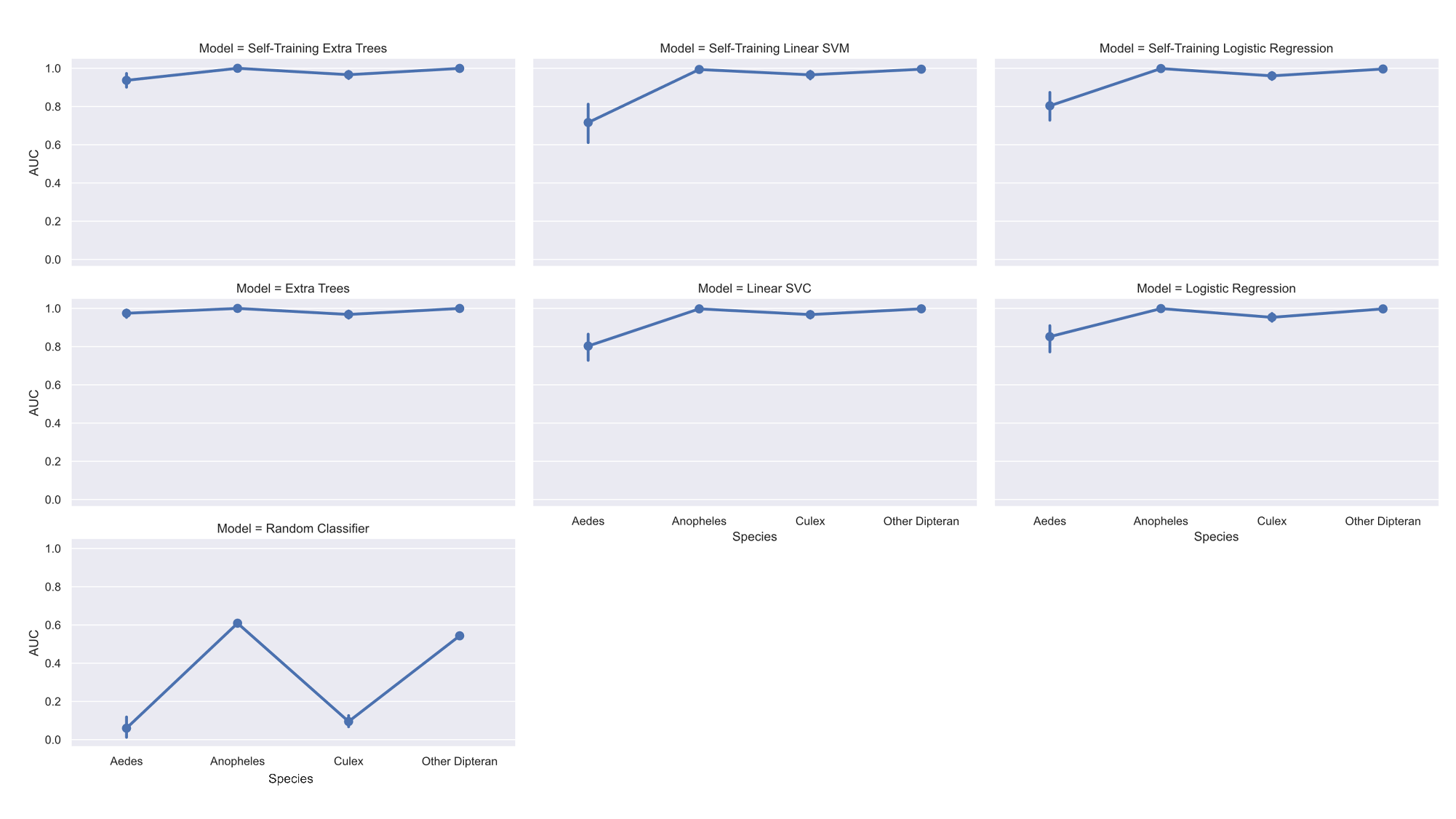


Supplementary Figure 6: Generalization performance of several semi-supervised and supervised models. The generalization performance score is the area under the curve (AUC) of a precision-recall curve. Twenty-five different curves were calculated for each species, and the average result and standard deviation is shown in the graphs above.

Table S.1. Listing of mosquito sequences used.

| **Genus** | **Species** | **Accession** |  |
| --- | --- | --- | --- |
| Aedeomyia | squamipennis* | OK662578 | |
| Aedes | aegypti | OM214531 | |
|  |  | EU352212 |  |
|  |  | MK575474 |  |
|  |  | MF194022 |  |
|  | alboannulatus | MN389464 |  |
|  |  | MN389465 |  |
|  | albopictus | AY072044 |  |
|  |  | KR068634 |  |
|  |  | MK575475 |  |
|  |  | KX383916 |  |
|  | alternans | MN389472 |  |
|  | flavopictus | MT501510 |  |
|  | koreicus | MT093832 |  |
|  | notoscriptus | KM676218 |  |
|  |  | KM676219 |  |
|  | rubrithorax | MN389466 |  |
| Anopheles | aconitus | KX887320 | |
|  | albertoi | MF381633 |  |
|  |  | MF381634 |  |
|  | albitarsis | MF381591 |  |
|  |  | MF381603 |  |
|  |  | MF381610 |  |
|  |  | MF381611 |  |
|  |  | MF381644 |  |
|  |  | MF381653 |  |
|  |  | MF381692 |  |
|  |  | MF381723 |  |
|  |  | MT757854 |  |
|  |  | HQ335349 |  |
|  |  | HQ335346 |  |
|  |  | HQ335344 |  |
|  |  | MW915567 |  |
|  |  | MZ062449 |  |
|  |  | MZ062450 |  |
|  |  | MZ062451 |  |
|  |  | MZ062452 |  |
|  |  | MZ062453 |  |
|  |  | MZ062454 |  |
|  |  | MZ062455 |  |
|  |  | MZ062456 |  |
|  |  | MZ062457 |  |
|  |  | MZ062458 |  |
|  |  | MZ062459 |  |
|  |  | MZ062471 |  |
|  |  | MZ062472 |  |
|  |  | MZ062473 |  |
|  |  | MZ062475 |  |
|  |  | MZ062476 |  |
|  | annulipes | MN389454 |  |
|  | anthropophagus* | MW279150 |  |
|  | antunesi | MF381676 |  |
|  |  | MF381735 |  |
|  | arabiensis | KT382816 |  |
|  | argyritarsis | MF381639 |  |
|  |  | MF381647 |  |
|  |  | MF381649 |  |
|  |  | MF381679 |  |
|  | arthuri | MF381636 |  |
|  |  | MF381712 |  |
|  | atacamensis | MF381600 |  |
|  | atroparvus | OU632726 |  |
|  |  | KT382817 |  |
|  | bellator | KU551287 |  |
|  | benarrochi | MF381584 |  |
|  |  | MF381586 |  |
|  |  | MF381588 |  |
|  | braziliensis | MF381598 |  |
|  |  | MF381732 |  |
|  | coluzzii | KT382819 |  |
|  | costai | MF381604 |  |
|  |  | MF381607 |  |
|  |  | MF381614 |  |
|  |  | MF381631 |  |
|  | coustani | MT806097 |  |
|  | cracens | JX219733 |  |
|  | cruzii | KU551284 |  |
|  |  | KU551285 |  |
|  |  | KU551286 |  |
|  |  | KU551289 |  |
|  |  | KJ701506 |  |
|  |  | MK575477 |  |
|  | culicifacies | KR732656 |  |
|  |  | KT382820 |  |
|  | darlingi | MF381589 |  |
|  |  | MF381596 |  |
|  |  | MF381608 |  |
|  |  | MF381626 |  |
|  |  | MF381650 |  |
|  |  | MF381671 |  |
|  |  | MF381675 |  |
|  |  | MF381713 |  |
|  |  | MF381725 |  |
|  |  | MF381726 |  |
|  |  | MF381728 |  |
|  |  | MF381733 |  |
|  |  | GQ918272 |  |
|  |  | GQ918273 |  |
|  |  | MK575478 |  |
|  | deaneorum | MF381590 |  |
|  |  | MF381654 |  |
|  |  | HQ335347 |  |
|  |  | MZ062460 |  |
|  |  | MZ062462 |  |
|  | dirus | JX219731 |  |
|  |  | KT899887 |  |
|  | eiseni | MF381678 |  |
|  |  | MF381706 |  |
|  | epiroticus | KT382821 |  |
|  | evansae | MF381609 |  |
|  |  | MF381667 |  |
|  |  | MF381707 |  |
|  |  | MF381711 |  |
|  |  | MF381734 |  |
|  | fluminensis | MF381677 |  |
|  |  | MF381699 |  |
|  | forattinii | MF381664 |  |
|  | funestus | MG742193 |  |
|  |  | MG742159 |  |
|  |  | MG742171 |  |
|  |  | MG742166 |  |
|  |  | MG742190 |  |
|  |  | MG742181 |  |
|  |  | MG742178 |  |
|  |  | MG742188 |  |
|  |  | MG742184 |  |
|  |  | MG742158 |  |
|  |  | MG742161 |  |
|  |  | MG742198 |  |
|  |  | MG742174 |  |
|  |  | MG742162 |  |
|  |  | MG742175 |  |
|  |  | MG742176 |  |
|  |  | MG742197 |  |
|  |  | MG742163 |  |
|  |  | MG742177 |  |
|  |  | MG742199 |  |
|  |  | MG742179 |  |
|  |  | MG742157 |  |
|  |  | MG742172 |  |
|  |  | MG742183 |  |
|  |  | MG742187 |  |
|  |  | MG742164 |  |
|  |  | MG742173 |  |
|  |  | MG742167 |  |
|  |  | MG742160 |  |
|  |  | MG742168 |  |
|  |  | MG742165 |  |
|  |  | MG742182 |  |
|  |  | MG742180 |  |
|  |  | MG742169 |  |
|  |  | MG742189 |  |
|  |  | MG742186 |  |
|  |  | MG742170 |  |
|  |  | MG742194 |  |
|  |  | MG742195 |  |
|  |  | MG742191 |  |
|  |  | MG742185 |  |
|  |  | MG742196 |  |
|  |  | MG742192 |  |
|  |  | DQ146364 |  |
|  |  | MT917167 |  |
|  |  | MT917168 |  |
|  |  | MT917169 |  |
|  |  | MT917170 |  |
|  |  | MT917171 |  |
|  |  | MT917172 |  |
|  |  | MT917173 |  |
|  |  | MT917174 |  |
|  |  | MT917175 |  |
|  |  | MT917176 |  |
|  |  | MT917177 |  |
|  |  | MT917178 |  |
|  |  | MT917179 |  |
|  |  | MT917180 |  |
|  |  | MT917181 |  |
|  |  | MT917182 |  |
|  |  | MF775371 |  |
|  | galvaoi | MF381666 |  |
|  |  | MF381669 |  |
|  |  | MF381719 |  |
|  | gambiae | L20934 |  |
|  |  | MG930827 |  |
|  |  | MG930828 |  |
|  |  | MG930829 |  |
|  |  | MG930830 |  |
|  |  | MG930834 |  |
|  |  | MG930869 |  |
|  |  | MG930886 |  |
|  |  | MG930888 |  |
|  |  | MG930871 |  |
|  |  | MG930883 |  |
|  | gilesi | MF381630 |  |
|  | goeldii | MF381656 |  |
|  |  | MF381657 |  |
|  | guarani | MF381674 |  |
|  | hinesorum | JX219734 |  |
|  | homunculus | MF381605 |  |
|  |  | MF381697 |  |
|  |  | MF381729 |  |
|  | ininii | MT757855 |  |
|  | janconnae | HQ335348 |  |
|  |  | MZ062464 |  |
|  |  | MZ062465 |  |
|  |  | MZ062466 |  |
|  |  | MZ062477 |  |
|  |  | MZ062478 |  |
|  | kompi | MF381721 |  |
|  | laneanus | MF381613 |  |
|  |  | KU551288 |  |
|  | lanei | MF381621 |  |
|  | longipalpis | MT917148 |  |
|  |  | MT917149 |  |
|  |  | MT917150 |  |
|  |  | MT917151 |  |
|  |  | MT917152 |  |
|  |  | MT917153 |  |
|  |  | MT917154 |  |
|  |  | MT917155 |  |
|  |  | MT917156 |  |
|  |  | MT917157 |  |
|  | lutzii | MF381701 |  |
|  |  | MF381702 |  |
|  | marajoara | MF381592 |  |
|  |  | MF381593 |  |
|  |  | MF381646 |  |
|  |  | MF381658 |  |
|  |  | MT588297 |  |
|  |  | MZ062479 |  |
|  |  | MZ062480 |  |
|  | medialis | MF381595 |  |
|  |  | MF381700 |  |
|  | melas | KT382823 |  |
|  | merus | KT382824 |  |
|  | minimus | KT895423 |  |
|  |  | KT382825 |  |
|  | minor | MF381627 |  |
|  |  | MF381684 |  |
|  | oryzalimnetes | MF381583 |  |
|  |  | MF381623 |  |
|  |  | MF381628 |  |
|  |  | MF381648 |  |
|  |  | MF381668 |  |
|  |  | HQ335345 |  |
|  |  | MZ062467 |  |
|  |  | MZ062469 |  |
|  |  | MZ062481 |  |
|  | oswaldoi | MF381602 |  |
|  |  | MF381624 |  |
|  |  | MF381704 |  |
|  | parensis | MT917138 |  |
|  |  | MT917139 |  |
|  |  | MT917140 |  |
|  |  | MT917141 |  |
|  |  | MT917142 |  |
|  |  | MT917143 |  |
|  |  | MT917144 |  |
|  |  | MT917145 |  |
|  |  | MT917146 |  |
|  |  | MT917147 |  |
|  | parvus | MF381635 |  |
|  |  | MF381645 |  |
|  |  | MF381670 |  |
|  | peryassui | MF381597 |  |
|  |  | MF381661 |  |
|  |  | MF381690 |  |
|  | pristinus | MF381716 |  |
|  | pseudotibiamaculatus | MF381737 |  |
|  | punctulatus | KT382826 |  |
|  | quadrimaculatus | MSQNCATR |  |
|  | rangeli | MF381582 |  |
|  |  | MF381686 |  |
|  | rondoni | MF381672 |  |
|  |  | MF381727 |  |
|  |  | MF381731 |  |
|  | sacharovi | MZ382473 |  |
|  |  | MZ382513 |  |
|  |  | MZ382474 |  |
|  |  | MZ382505 |  |
|  |  | MZ382478 |  |
|  |  | MZ382485 |  |
|  |  | MZ382509 |  |
|  |  | MZ382488 |  |
|  |  | MZ382508 |  |
|  |  | MZ382510 |  |
|  |  | MZ382516 |  |
|  |  | MZ382489 |  |
|  |  | MZ382475 |  |
|  |  | MZ382479 |  |
|  |  | MZ382490 |  |
|  |  | MZ382494 |  |
|  |  | MZ382512 |  |
|  |  | MZ382497 |  |
|  |  | MZ382524 |  |
|  |  | MZ382514 |  |
|  |  | MZ382495 |  |
|  |  | MZ382500 |  |
|  |  | MZ382491 |  |
|  |  | MZ382501 |  |
|  |  | MZ382544 |  |
|  |  | MZ382536 |  |
|  |  | MZ382543 |  |
|  |  | MZ382533 |  |
|  |  | MZ382535 |  |
|  |  | MZ382545 |  |
|  |  | MZ382519 |  |
|  |  | MZ382517 |  |
|  |  | MZ382526 |  |
|  |  | MZ382531 |  |
|  |  | MZ382532 |  |
|  |  | MW366634 |  |
|  |  | MZ382529 |  |
|  |  | MZ382499 |  |
|  |  | MZ382530 |  |
|  |  | MZ382492 |  |
|  |  | MZ382498 |  |
|  |  | MZ382521 |  |
|  |  | MZ382480 |  |
|  |  | MZ382486 |  |
|  |  | MZ382525 |  |
|  |  | MZ382515 |  |
|  |  | MZ382496 |  |
|  |  | MZ382493 |  |
|  |  | MZ382537 |  |
|  |  | MZ382528 |  |
|  |  | MZ382539 |  |
|  |  | MZ382540 |  |
|  |  | MZ382520 |  |
|  |  | MZ382527 |  |
|  |  | MZ382538 |  |
|  |  | MZ382541 |  |
|  |  | MZ382523 |  |
|  |  | MZ382518 |  |
|  |  | MZ382522 |  |
|  |  | MZ382542 |  |
|  |  | MZ382534 |  |
|  |  | MZ382502 |  |
|  |  | MZ382477 |  |
|  |  | MZ382504 |  |
|  |  | MZ382481 |  |
|  |  | MZ382482 |  |
|  |  | MZ382483 |  |
|  |  | MZ382484 |  |
|  |  | MZ382506 |  |
|  |  | MZ382511 |  |
|  |  | MZ382487 |  |
|  |  | MZ382507 |  |
|  |  | MZ382476 |  |
|  |  | MZ382503 |  |
|  | sawyeri | MF381617 |  |
|  |  | MF381632 |  |
|  |  | MF381643 |  |
|  | sinensis | MG816533 |  |
|  |  | MG816534 |  |
|  |  | MG816535 |  |
|  |  | MG816536 |  |
|  |  | MG816538 |  |
|  |  | MG816539 |  |
|  |  | MG816540 |  |
|  |  | MG816541 |  |
|  |  | MG816542 |  |
|  |  | MG816543 |  |
|  |  | MG816544 |  |
|  |  | MG816546 |  |
|  |  | MG816547 |  |
|  |  | MG816548 |  |
|  |  | MG816549 |  |
|  |  | MG816550 |  |
|  |  | MG816551 |  |
|  |  | MG816552 |  |
|  |  | MG816553 |  |
|  |  | MG816554 |  |
|  |  | MG816555 |  |
|  |  | MG816556 |  |
|  |  | MG816557 |  |
|  |  | MG816558 |  |
|  |  | MG816559 |  |
|  |  | MG816561 |  |
|  |  | MG816562 |  |
|  |  | MG816563 |  |
|  |  | MG816564 |  |
|  |  | MG816566 |  |
|  |  | MG816567 |  |
|  |  | MG816568 |  |
|  |  | MF322628 |  |
|  |  | OK458560 |  |
|  | splendidus | KX887321 |  |
|  | stephensi | KT899888 |  |
|  |  | KT382827 |  |
|  | striatus | MF381625 |  |
|  |  | MF381637 |  |
|  | strodei | MF381640 |  |
|  |  | MF381698 |  |
|  |  | MF381724 |  |
|  | triannulatus | MF381622 |  |
|  |  | MF381629 |  |
|  |  | MF381638 |  |
|  |  | MF381651 |  |
|  |  | MF381730 |  |
|  | vaneedeni | MT917128 |  |
|  |  | MT917129 |  |
|  |  | MT917130 |  |
|  |  | MT917131 |  |
|  |  | MT917132 |  |
|  |  | MT917133 |  |
|  |  | MT917134 |  |
|  |  | MT917135 |  |
|  |  | MT917136 |  |
|  |  | MT917137 |  |
| Bironella | hollandi | MF381612 | |
| Coquillettidia | chrysonotum | MK575479 | |
|  | linealis | MN389455 |  |
| Culex | annulirostris | MN389469 | |
|  | australicus | MN389456 |  |
|  | bidens | MF381652 |  |
|  | bilineatus | MF381693 |  |
|  |  | MF381722 |  |
|  | brami | MF381736 |  |
|  | camposi | MF040164 |  |
|  | chidesteri | MF381720 |  |
|  | coronator | MF381691 |  |
|  |  | MF509887 |  |
|  |  | MF509895 |  |
|  |  | MF509888 |  |
|  |  | MF509889 |  |
|  | cylindricus | MN389457 |  |
|  | declarator | MF381714 |  |
|  | fergusoni | MN389458 |  |
|  | gelidus | KX753344 |  |
|  | lygrus | MF381718 |  |
|  | mollis | MF381660 |  |
|  |  | MF381705 |  |
|  | nigripalpus | MF381715 |  |
|  | orbostiensis | MN389461 |  |
|  | pipiens | MN389459 |  |
|  |  | MN389460 |  |
|  |  | KT851543 |  |
|  | quinquefasciatus | MN389462 |  |
|  |  | GU188856 |  |
|  |  | MK575480 |  |
|  | sitiens | MN389463 |  |
|  | surinamensis | MF381615 |  |
|  | tritaeniorhynchus | KT852976 |  |
|  | usquatissimus | MF040165 |  |
|  |  | MF509891 |  |
|  |  | MF509893 |  |
|  |  | MF509894 |  |
|  |  | MF040163 |  |
|  | usquatus | MF040161 |  |
| Limatus | flavisetosus | MK575482 | |
| Lutzia | fuscana | MH316118 | |
|  | halifaxii | MH316119 |  |
| Mansonia | amazonensis | MK575483 | |
|  | uniformis | MN342085 |  |
| Ochlerotatus | fulvus | MK575476 | |
|  | nigrithorax | MN389467 |  |
|  | taeniorhynchus | MN626442 |  |
|  | vigilax | KP721463 |  |
|  |  | MK575484 |  |
|  | vittiger | MN389473 |  |
| Psorophora | albipes | OK662581 | |
|  | ferox | OK662582 |  |
|  |  | MK575485 |  |
| Runchomyia | reversa | MK575487 | |
| Sabethes | belisarioi | MF957171 | |
|  | chloropterus | MF957172 |  |
|  | glaucodaemon | MF957173 |  |
|  | undosus | MK575488 |  |
| Trichoprosopon | pallidiventer | MK575490 | |
| Tripteroides | tasmaniensis | MN389468 | |
| Uranotaenia | geometrica | MK575491 | |
| Wyeomyia | confusa | MK575492 | |

*These species are not recognised by the Mosquito Taxonomic Inventory as valid mosquito species as of 2022-11-17.

Table S.2. Listing of non-Culicidae Dipteran sequences used.

| **Genus** | **Species** | **Accession** |
| --- | --- | --- |
| *Anastrepha* | *fraterculus* | KX926433 |
| *Apocephalus* | *antennatus* | MG546669 |
| *Arachnocampa* | *flava* | JN861748 |
| *Archisepsis* | *discolor* | KT272843 |
| *Atylotus* | *miser* | KT225291 |
| *Bactrocera* | *arecae* | KR233259 |
|  | *biguttula* | MK293875 |
|  | *carambolae* | EF014414 |
|  | *caudata* | KT625491 |
|  |  | KT625492 |
|  | *correcta* | JX456552 |
|  | *cucurbitae* | JN635562 |
|  | *depressa* | KY131831 |
|  | *diaphora* | KT159730 |
|  | *dorsalis* | DQ845759 |
|  |  | DQ917577 |
|  |  | KM244662 |
|  |  | KT343905 |
|  | *invadens* | KX534207 |
|  | *latifrons* | KT881556 |
|  | *limbifera* | MG566056 |
|  | *melastomatos* | KT881557 |
|  | *minax* | HM776033 |
|  | *oleae* | AY210702 |
|  |  | AY210703 |
|  |  | GU108459 |
|  |  | GU108460 |
|  |  | GU108461 |
|  |  | GU108462 |
|  |  | GU108463 |
|  |  | GU108464 |
|  |  | GU108465 |
|  |  | GU108466 |
|  |  | GU108467 |
|  |  | GU108468 |
|  |  | GU108469 |
|  |  | GU108470 |
|  |  | GU108471 |
|  |  | GU108472 |
|  |  | GU108473 |
|  |  | GU108474 |
|  |  | GU108475 |
|  |  | GU108476 |
|  |  | GU108477 |
|  |  | GU108478 |
|  |  | GU108479 |
|  |  | KR677101 |
|  |  | KR677102 |
|  |  | KR677103 |
|  |  | KR677104 |
|  |  | KR677105 |
|  |  | KR677106 |
|  |  | KR677107 |
|  | *papayae* | DQ917578 |
|  | *philippinensis* | DQ995281 |
|  | *ritsemai* | MF668132 |
|  | *scutellata* | KT159731 |
|  | *tryoni* | HQ130030 |
|  | *tsuneonis* | MF540918 |
|  | *umbrosa* | KT881558 |
|  | *zonata* | KP296150 |
| *Bittacomorphella* | *fenderiana* | JN861745 |
| *Blepharipa* | *sp. CMERI-Uzi-001* | KY644698 |
| *Calliphora* | *vicina* | JX913760 |
|  | *vomitoria* | KT444440 |
| *Ceratitis* | *capitata* | AJ242872 |
|  | *fasciventris* | KY436396 |
| *Cestrotus* | *liui* | KX372559 |
| *Chironomus* | *tepperi* | JN861749 |
| *Chromatomyia* | *horticola* | KR047789 |
| *Chrysomya* | *albiceps* | JX913736 |
|  | *bezziana* | JX913737 |
|  | *megacephala* | KT272775 |
|  | *nigripes* | KT444441 |
|  | *phaonis* | KX500359 |
|  | *pinguis* | KM244730 |
|  | *putoria* | AF352790 |
|  | *rufifacies* | JX913740 |
|  |  | JX913741 |
| *Clemelis* | *pullata* | MG838887 |
| *Cochliomyia* | *hominivorax* | AF260826 |
| *Cramptonomyia* | *spenceri* | JN861747 |
| *Cydistomyia* | *duplonotata* | DQ866052 |
| *Cylindrotoma* | *sp. ZK-2016* | KT970060 |
| *Dacus* | *conopsoides* | MH351199 |
|  | *longicornis* | KX345846 |
| *Delia* | *antiqua* | KT026595 |
| *Dermatobia* | *hominis* | AY463155 |
| *Dixella* | *aestivalis* | KT878382 |
|  | *sp. ZK-2014* | KM245574 |
| *Drosophila* | *albomicans* | KT119344 |
|  | *americana* | HQ849834 |
|  | *borealis* | HQ849822 |
|  |  | HQ849823 |
|  | *canadiana* | HQ849825 |
|  | *ezoana* | HQ849832 |
|  | *flavomontana* | HQ849829 |
|  | *guanche* | LS398100 |
|  | *incompta* | KM275233 |
|  | *kanekoi* | HQ849827 |
|  | *lacicola* | HQ849821 |
|  | *littoralis* | FJ447340 |
|  |  | HQ849824 |
|  | *lummei* | HQ849833 |
|  | *melanica* | HQ849828 |
|  | *melanogaster* | DMU37541 |
|  |  | JQ686693 |
|  |  | JQ686694 |
|  |  | KJ947872 |
|  |  | KP161877 |
|  |  | KP843842 |
|  |  | KP843843 |
|  |  | KP843844 |
|  |  | KP843845 |
|  |  | KP843846 |
|  |  | KP843847 |
|  |  | KP843849 |
|  |  | KP843851 |
|  |  | KP843852 |
|  |  | KP843854 |
|  |  | KT174472 |
|  |  | KT174473 |
|  |  | KT174474 |
|  |  | KU764535 |
|  | *montana* | HQ849820 |
|  | *novamexicana* | HQ849830 |
|  | *robusta* | HQ849826 |
|  | *santomea* | KF824856 |
|  |  | KF824857 |
|  |  | KF824858 |
|  |  | KF824859 |
|  |  | KF824860 |
|  |  | KF824861 |
|  |  | KF824862 |
|  |  | KF824863 |
|  |  | KF824864 |
|  |  | KF824865 |
|  |  | KF824866 |
|  |  | KF824867 |
|  |  | KF824868 |
|  |  | KF824869 |
|  |  | KF824870 |
|  |  | KF824871 |
|  |  | KF824872 |
|  | *suzukii* | KU588141 |
|  | *virilis* | HQ849831 |
|  | *yakuba* | KF824873 |
|  |  | KF824874 |
|  |  | KF824875 |
|  |  | KF824876 |
|  |  | KF824877 |
|  |  | KF824878 |
|  |  | KF824879 |
|  |  | KF824880 |
|  |  | KF824881 |
|  |  | KF824882 |
|  |  | KF824883 |
|  |  | KF824884 |
|  |  | KF824885 |
|  |  | KF824886 |
|  |  | KF824887 |
|  |  | KF824888 |
|  |  | KF824889 |
|  |  | KF824890 |
|  |  | KF824891 |
|  |  | KF824892 |
|  |  | KF824893 |
|  |  | KF824894 |
|  |  | KF824895 |
|  |  | KF824896 |
|  |  | KF824897 |
|  |  | KF824898 |
|  |  | KF824899 |
|  |  | KF824900 |
|  |  | KF824901 |
|  |  | X03240 |
| *Episyrphus* | *balteatus* | KU351241 |
| *Eristalinus* | *aeneus* | MH321208 |
|  | *barclayi* | MH321205 |
|  | *fuscicornis* | MH321204 |
|  | *tabanoides* | MH321207 |
|  | *vicarians* | MH321206 |
| *Eristalis* | *tenax* | MH159199 |
| *Eupeodes* | *corollae* | KU379658 |
| *Euryomma* | *sp. SMD-2015* | KP901269 |
| *Evandromyia* | *infraspinosa* | KX356033 |
| *Exorista* | *civilis* | MG792804 |
|  | *japonica* | MK903727 |
| *Fergusonina* | *sp. LAN-2011* | HQ872008 |
| *Forcipomyia* | *sp. m QL-2019* | MK000395 |
| *Fucellia* | *costalis* | MH823369 |
| *Gasterophilus* | *intestinalis* | MG920504 |
|  | *nasalis* | MG920505 |
|  | *pecorum* | KU578262 |
| *Graphomya* | *rufitibia* | MG735216 |
| *Gyrostigma* | *rhinocerontis* | MK045312 |
| *Haematobia* | *irritans* | DQ029097 |
|  |  | KM669714 |
| *Hemipyrellia* | *ligurriens* | JX913759 |
| *Hermetia* | *illucens* | KY679159 |
| *Heterostomus* | *sp. SMD-2018* | MH817480 |
| *Hydrotaea* | *capensis* | MH973639 |
|  | *chalcogaster* | MH521131 |
|  | *ignava* | KY977435 |
|  | *leucostoma* | KY977434 |
|  | *sp. ACMJ-2016* | KT272841 |
|  | *spinigera* | MH705623 |
| *Hypoderma* | *lineatum* | GU584123 |
| *Limonia* | *phragmitidis* | MK673118 |
| *Liriomyza* | *bryoniae* | JN570504 |
|  | *chinensis* | MG252777 |
|  | *huidobrensis* | JN570505 |
|  |  | JQ862474 |
|  | *sativae* | HQ333260 |
|  |  | JQ862475 |
|  | *trifolii* | GU327644 |
|  |  | JN570506 |
| *Lucilia* | *coeruleiviridis* | KT272780 |
|  | *cuprina* | JX913745 |
|  |  | JX913746 |
|  |  | JX913747 |
|  |  | JX913748 |
|  |  | JX913750 |
|  |  | JX913751 |
|  |  | JX913752 |
|  |  | JX913753 |
|  | *porphyrina* | JX913758 |
|  | *sericata* | JX913754 |
|  |  | JX913756 |
|  |  | JX913757 |
|  |  | KT272854 |
| *Lutzomyia* | *trinidadensis* | KX356037 |
| *Mayetiola* | *destructor* | GQ387648 |
| *Megaselia* | *scalaris* | KF974742 |
| *Melanagromyza* | *sojae* | KT597923 |
| *Melophagus* | *ovinus* | KX870852 |
|  |  | MH024396 |
| *Micropygomyia* | *cayennensis* | KX356032 |
| *Musca* | *domestica* | KM200723 |
|  |  | KT444442 |
|  | *sorbens* | MG941012 |
|  |  | MH521132 |
| *Muscina* | *angustifrons* | KY495724 |
|  | *stabulans* | KM676394 |
| *Nemopoda* | *mamaevi* | KM605250 |
| *Nemorilla* | *maculosa* | MG786426 |
| *Neoceratitis* | *asiatica* | MF434829 |
| *Orseolia* | *oryzae* | KM888183 |
| *Oxysarcodexia* | *avuncula* | MH879754 |
|  | *terminalis* | MH879757 |
|  | *thornax* | MH879753 |
|  |  | MH879756 |
|  |  | MH879765 |
|  | *varia* | MH879764 |
| *Pachycerina* | *decemlineata* | KX372561 |
| *Palesisa* | *nudioculata* | MH256563 |
| *Paracladura* | *trichoptera* | JN861751 |
| *Parochlus* | *steinenii* | KT003702 |
| *Peckia* | *australis* | MH879762 |
|  | *collusor* | MH879763 |
|  | *resona* | MH879760 |
|  |  | MH879761 |
| *Phlebotomus* | *chinensis* | KR349297 |
|  | *papatasi* | KR349298 |
| *Phormia* | *regina* | KT272842 |
|  |  | KX853042 |
| *Pressatia* | *choti* | KX356038 |
| *Procecidochares* | *utilis* | KC355248 |
| *Protophormia* | *terraenovae* | JX913743 |
| *Protoplasa* | *fitchii* | JN861746 |
| *Psathyromyia* | *aragaoi* | KX356042 |
| *Ptychoptera* | *sp. ATB-2011* | JN861744 |
| *Ravinia* | *pernix* | KM676414 |
| *Rhopalomyia* | *pomum* | GQ387649 |
| *Rutilia* | *goerlingiana* | JX913762 |
| *Sarcophaga* | *africa* | KM881633 |
|  | *antilope* | MH540748 |
|  | *dux* | MH540745 |
|  |  | MH937748 |
|  | *impatiens* | JN859549 |
|  | *melanura* | KP091687 |
|  |  | MH879758 |
|  | *princeps* | MH981944 |
|  | *ruficornis* | MH937749 |
|  | *similis* | KM287431 |
| *Scathophaga* | *stercoraria* | KM200724 |
| *Sciopemyia* | *fluviatilis* | KX356039 |
| *Simosyrphus* | *grandicornis* | DQ866050 |
| *Simulium* | *aureohirtum* | KP793690 |
|  | *maculatum* | MH729190 |
|  | *variegatum* | KU252587 |
| *Spaniocelyphus* | *pilosus* | KX372562 |
| *Stomoxys* | *calcitrans* | DQ533708 |
|  |  | KT272851 |
| *Sylvicola* | *fenestralis* | JN861752 |
| *Symplecta* | *hybrida* | KT970064 |
| *Synthesiomyia* | *nudiseta* | MK007046 |
| *Trichocera* | *bimacula* | JN861750 |
| *Trichophthalma* | *punctata* | DQ866051 |
| *Trichopygomyia* | *trichopyga* | KX356035 |
| *Viannamyia* | *furcata* | KX356034 |
| *Zeugodacus* | *scutellatus* | MF358969 |
|  | *tau* | MF966383 |
|  |  | MF966384 |
